# Chemical Prelithiated 3D Lithiophilic/-Phobic Interlayer Enables Long-Term Li Plating/Stripping

**DOI:** 10.1021/acsnano.4c04507

**Published:** 2024-06-28

**Authors:** Sandro Schöner, Dana Schmidt, Xinchang Chen, Krzysztof Dzieciol, Roland Schierholz, Pengfei Cao, Ahmad Ghamlouche, Fabian Jeschull, Anna Windmüller, Chih-Long Tsai, Xunfan Liao, Hans Kungl, Gui-Ming Zhong, Yiwang Chen, Hermann Tempel, Shicheng Yu, Rüdiger-A. Eichel

**Affiliations:** † Institute of Energy and Climate Research (IEK-9: Fundamental Electrochemistry), Forschungszentrum Jülich, 52428 Jülich, Germany; ‡ Institut für Materialien und Prozesse für elektrochemische Energiespeicher und wandler, RWTH Aachen University, 52074 Aachen, Germany; § Laboratory of Advanced Spectro-electrochemistry and Li-Ion Batteries, Dalian Institute of Chemical Physics, Chinese Academy of Sciences, Dalian 116023, China; ∥ Ernst Ruska-Centre for Microscopy and Spectroscopy with Electrons, Forschungszentrum Jülich, 52428 Jülich, Germany; ⊥ Karlsruher Institute of Technologie (KIT), Institute for Applied Materials-Energy Storage Systems (IAM-ESS), 76344 Eggenstein Leopoldshafen, Germany; ○ National Engineering Research Center for Carbohydrate Synthesis/Key Lab of Fluorine and Silicon for Energy Materials and Chemistry of Ministry of Education, Jiangxi Normal University, 330022 Nanchang, China

**Keywords:** prelithiation, zero-excess Li metal batteries, anode-free, anode-less, carbon fibers, lithiophilic−lithiophobic gradient

## Abstract

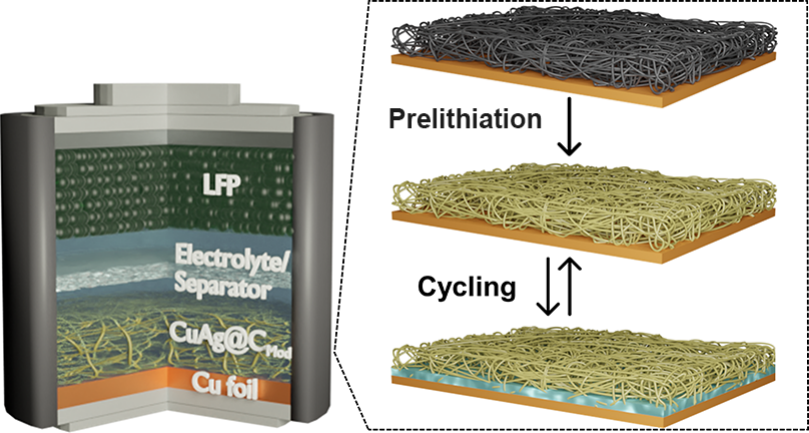

The up-to-date lifespan of zero-excess lithium (Li) metal batteries is limited to a few dozen cycles due to irreversible Li-ion loss caused by interfacial reactions during cycling. Herein, a chemical prelithiated composite interlayer, made of lithiophilic silver (Ag) and lithiophobic copper (Cu) in a 3D porous carbon fiber matrix, is applied on a planar Cu current collector to regulate Li plating and stripping and prevent undesired reactions. The Li-rich surface coating of lithium oxide (Li_2_O), lithium carboxylate (RCO_2_Li), lithium carbonates (ROCO_2_Li), and lithium hydride (LiH) is formed by soaking and directly heating the interlayer in *n*-butyllithium hexane solution. Although only a thin coating of ∼10 nm is created, it effectively regulates the ionic and electronic conductivity of the interlayer via these surface compounds and reduces defect sites by reactions of *n*-butyllithium with heteroatoms in the carbon fibers during formation. The spontaneously formed lithiophilic–lithiophobic gradient across individual carbon fiber provides homogeneous Li-ion deposition, preventing concentrated Li deposition. The porous structure of the composite interlayer eliminates the built-in stress upon Li deposition, and the anisotropically distributed carbon fibers enable uniform charge compensation. These features synergistically minimize the side reactions and compensate for Li-ion loss while cycling. The prepared zero-excess Li metal batteries could be cycled 300 times at 1.17 C with negligible capacity fading.

## Introduction

Because of the continuously growing demand for higher energy density Li-ion batteries, a so-called zero-excess Li metal battery (or anode-free battery) with theoretically high energy density has received widespread attention.^1^ As early as 2000, such a concept was described by Neudecker et al. as a “Li-free” battery with an *in situ* plated Li anode.^2^ Later, several terms were proposed, such as anode-free, anode-less, or zero-excess battery.^3−7^ However, as Hatzell pointed out, these definitions lead to some discussions and considerations.^8^ Here, the “zero-excess Li metal battery”, where Li metal is formed on the current collector during charging, is adopted to describe the cell configuration. In such a design, Li-ions are deposited from the cathode directly on a planar negative current collector (mostly Cu foil), resulting in improved safety due to the limited amount of metallic Li and significantly increased energy density compared with conventional Li-ion and Li metal batteries.^9,10^ Besides, the manufacturing process is simplified, and the costs for fabricating these batteries are reduced, as no complicated anode preparation is required in theory.

Practically, challenges remain in extending the lifespan of such zero-excess Li metal batteries. State-of-the-art studies indicate the cycling life of zero-excess Li metal batteries is limited to about 100 cycles with a maximum 80% capacity retention.^11−13^ The associated low Coulombic efficiency (CE; ≤99%) indicates a continuous loss of lithium during cycling due to the formation of mossy/dead Li caused by nonuniform Li deposition on the lithiophobic Cu foil current collector^14^ and the formation–deformation–reformation of the solid electrolyte interface (SEI), especially during initial cycles.^15−17^ Additionally, the volume change on the anode side in zero-excess Li metal batteries could build up strong local stress during cycling, forming mossy and dead Li in the case of crack growth or Li particle loss. Overall, maximizing capacity retention and achieving CE above 99.95% via avoiding the above-mentioned side reactions are the keys to improving the cycle life of zero-excess Li metal batteries.^18^

Current collector modification is one of the promising approaches to sustaining the limited Li-ion inventory in zero-excess Li metal batteries and stabilizing the Li deposition/dissolution via homogeneous Li-ion flux while cycling.^19,20^ Since the overpotential for nucleating metallic Li is critical for improving the density and uniformity of Li deposition, lithiophilic substrates such as Ag and magnesium have been used as the current collector to suppress the nucleation overpotential for metallic Li, thereby avoiding the formation of dead Li and resulting in a more homogeneous Li deposition.^10,11^ Accordingly, a lithiophilic–lithiophobic composite interlayer has been introduced, which consists of lithiophobic carbon nano tubes and lithiophilic zinc oxide on metallic Li anode, to suppress Li dendrites and enable long-term cyclability.^21^ Due to a negative Gibbs formation energy between the interlayer and Li, the Li nucleation first occurs at the interface between the substance and metallic Li.^22^ The deposited Li then firmly connects the whole interlayer, thereby preventing the formation of Li dendrites. The lithiophobic part is energetically averse to the Li nucleation and, therefore, acts as a protective layer between the deposited Li and the separator, avoiding concentrated Li deposition and short circuits.^21^

SEI formation during initial cycles consumes Li-ions caused by electrolyte–Li decomposition reactions on the anode side.^23^ For zero-excess Li metal batteries, due to the limited Li inventory in the system, such consumption of Li-ions for SEI formation seems to be minimized only by chemical prelithiation of the anode or current collector. This relies on the redox reactions between the lithiation agent and the relevant anode materials/current collector to insert a certain amount of Li-ions into their structure. In 1998, Scott et al. introduced chemical prelithiation of carbon black by immersing it in a *n*-butyllithium (*n*-BuLi) hexane solution to reduce the initial irreversible capacity loss while cycling the batteries. Although high initial discharge capacity was achieved, the overall capacity retention is low due to the build-up surface coating by chemical lithiation on carbon black being thicker and more brittle than the electrochemical formed SEI, especially with prolonged *n*-BuLi soaking.^24^ Likely, this is caused by the slight redox potential difference between carbon black and *n*-BuLi (1 V vs Li/Li^+^), inhibiting the reduction of carbon black and preventing substantial Li-ion insertion into the structure. Hence, a better strategy to use *n*-BuLi as the chemical lithiation agent must be developed.

A 3D porous buffer layer on top of the current collector could efficiently eliminate volume change influences during cycling on the anode side in zero-excess Li metal batteries. The stress encountered at the interface of the deposited Li can be reduced, minimizing volumetric change and stabilizing the SEI. The Sand’s time model demonstrates that by using 3D layers, such as carbon fibers, the local current density at the current collector is decreased due to the enhanced surface area of 3D structures in comparison to planar current collectors, which suppresses the formation of Li dendrites and homogenize Li deposition.^25,26^ Rao et al. showed that using carbon fibers as current collector support could significantly enhance overall battery performance compared to hard carbon and graphite due to the larger specific surface area of carbon fibers, whereby more sites for Li nucleation are created.^27^ However, irreversible corrosion reactions between carbon materials and liquid electrolytes lead to low CE and rapid capacity loss during cycling.^28^ Such consumption of the Li-ion inventory significantly harms the cycling stability of zero-excess Li metal batteries.

Here, a 3D porous interlayer design is presented and tested on the Cu foil current collector to examine the Li deposition/dissolution behaviors under excess and limited Li-ion inventory conditions (*cf*. half cells and zero-excess Li metal batteries). The composite interlayer comprises embedded lithiophilic Ag and lithiophobic Cu in a carbon fiber matrix treated by a modified *n*-BuLi prelithiation method. The core-to-shell lithiophilic-lithiophobic gradient structure of the interlayer favors a homogeneous Li deposition around the carbon fibers facing the planar Cu current collector. The porous feature of the interlayer offers sufficient free volume for Li deposition on the lithiated carbon fibers, compensating for volumetric build-up stress during cycling. The anisotropically distributed carbon fibers enable uniform charge compensation to avoid concentrated charge transfer reactions. Chemical lithiation of the 3D porous interlayer forms a thin layer of Li compounds on the surface that reduces the Li-ion loss caused by the reaction of the carbon fiber matrix with the liquid electrolyte. Due to the synergistic effects of the above-mentioned strategies, the zero-excess Li metal batteries consisting of LiFePO_4_ (LFP) cathodes and prelithiated interlayers on Cu demonstrated long-term cyclability and high CE at different current densities. In addition, no formation cycle at all applied current densities is needed for the prepared zero-excess Li metal batteries. The samples can be directly cycled at different C-rates, even at 1.17 C. These results provide insights into enhancing the cycling life of zero-excess Li metal batteries with limited Li-ion inventory, that is, utilizing material design and pretreatment to eliminate and compensate for possible loss of Li-ions in the batteries.

## Results and Discussion

**Figure 1:**
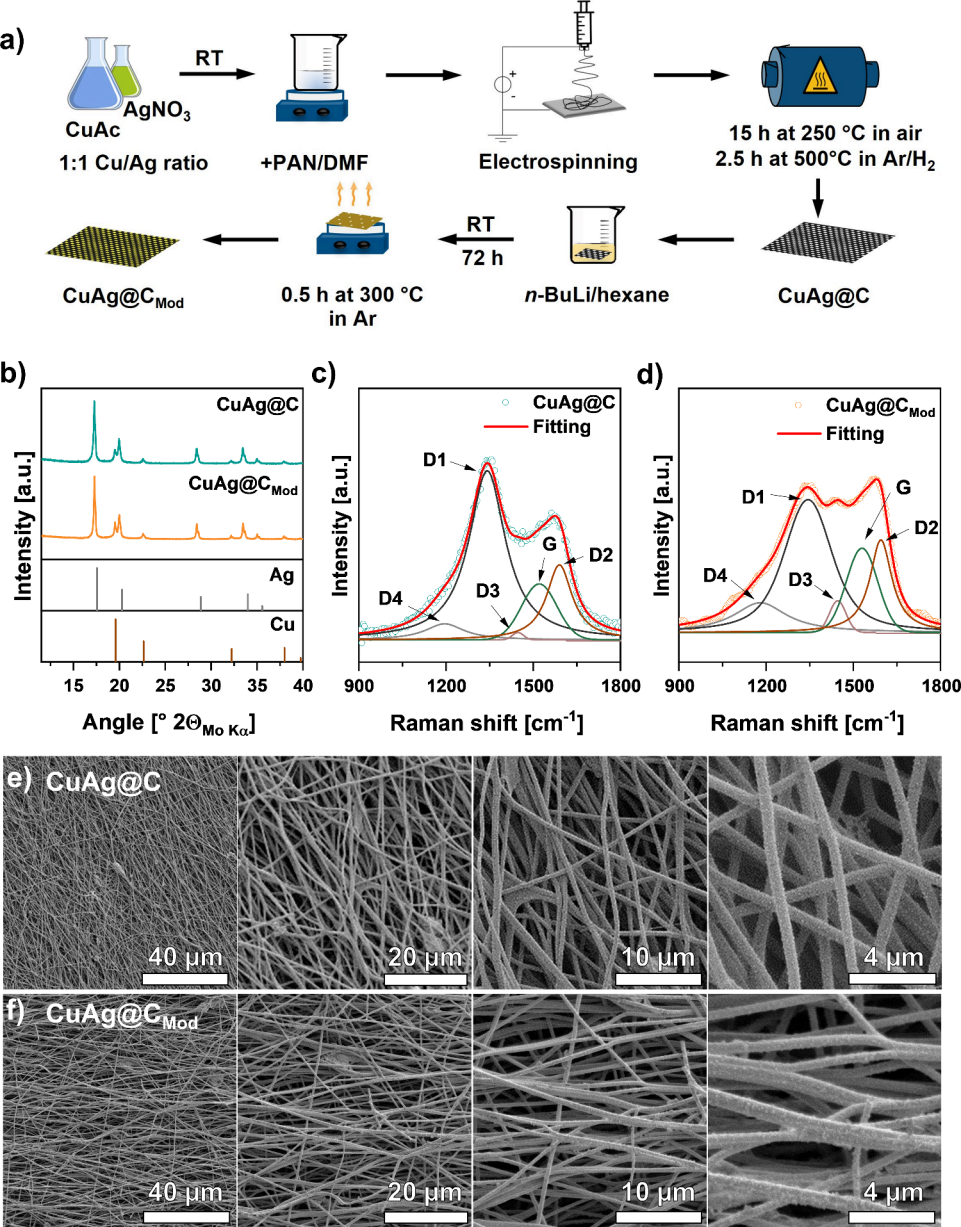
(a) Schematic illustration of the synthesis route of CuAg@C and the chemical prelithiation process. (b) XRD pattern of CuAg@C and CuAg@C_Mod_ measured with Mo–Kα radiation in transmission mode showing Cu and Ag reflections. The reference XRD patterns are Cu (ICSD 136042) and Ag (ICSD 22434). (c) Raman spectra of CuAg@C (left) and (d) CuAg@C_Mod_ (right) were measured in an ECC-Opto-Std cell to protect the sample from the ambient atmosphere. The fitting includes the band combination of the first-order Raman bands (G and D1–D4). (e) SEM images of CuAg@C (top) and (f) CuAg@C_Mod_ (bottom) illustrating a 3D porous feature.

The fabrication of the porous 3D composite of Cu and Ag particles on carbon fibers and the chemical prelithiation process are schematically illustrated in Figure 1a and described in detail in the Experimental Section. The pristine composite interlayers, namely, CuAg@C, based on polyacrylonitrile (PAN) with Cu and Ag particles embedded, were synthesized via electrospinning, followed by stabilization in air at 250 °C and carbonization/reduction step at 500 °C under Ar/H_2_ (97:3) flow. For chemical prelithiation, instead of conventional chemical lithiation of soaking and washing the samples in 2.5 M *n*-BuLi/hexane solution, CuAg@C was soaked in the solution for 3 days at room temperature and directly heated at 300 °C for 0.5 h in the glovebox after removing the excess *n*-BuLi solution. The heating process during prelithiation is expected to generate Li-containing redox products that benefit the compatibility of the composite interlayer with Li and electrolyte in the batteries. The lithiated sample, namely, CuAg@C_Mod_, and the pristine sample CuAg@C were then cut into desired sizes for characterization and battery assembly without further purification, saving time and resources. Meanwhile, the amount of lithium introduced into CuAg@C_Mod_ can be estimated by the amount of *n*-BuLi used for lithiation. During the prelithiation process before heating, the amount of 2.5 M *n*-BuLi/hexane solution added to the sample is 0.053 mL cm^–2^. After heat treatment at 300 °C for 0.5 h, Li-ions will not be removed, resulting in an approximately lithium areal density of 0.9137 mg cm^–2^ in the CuAg@C_Mod_.

The occurrence of lithiophobic Cu and lithiophilic Ag in CuAg@C and CuAg@C_Mod_ was proven by X-ray diffraction (XRD) measurements. Figure 1b shows the XRD pattern of CuAg@C and CuAg@C_Mod_ with the main reflections corresponding to Ag at 17.30°, 20.01°, 28.54°, and 33.57° and Cu at 19.57°, 22.60°, 32.20°, and 37.94°, respectively. Besides, the XRD patterns do not include any graphitic reflections, indicating a disordered carbon structure for both samples. A disordered carbon structure is beneficial for an isotropic Li-ion transport since the electron distribution is less concentrated than in highly ordered graphitic carbon, further reducing the probability of Li dendrite formation.^29^ The specific surface area of CuAg@C and CuAg@C_Mod_ was calculated using the Brunauer–Emmett–Teller (BET) method. Both samples show comparable results under Ar with a surface area of 7.2 m^2^ g^–1^ for CuAg@C and 4.9 m^2^ g^–1^ for CuAg@C_Mod_ (Figure S1) corresponding to the geometrical surface of the carbon fiber, indicating a small loss in roughness for the modified sample, most likely caused by the coating film. To analyze the microporous structure of the fibers, carbon dioxide (CO_2_) isotherm at 273 K has been recorded since argon or nitrogen could not access tiny pores (<0.7 nm) at cryogenic temperatures (Figure S2). Due to the applied electrospinning and heat treatment, an average pore volume of 0.058 cm^3^ g^–1^ and a much larger surface area of 209.1 m^2^ g^–1^ with a pore width of 0.37 nm is obtained by Monte Carlo calculations for CuAg@C, indicating a microporous carbon structure of the interlayer (Figure S3). Due to the highly reactive coating layer on CuAg@C_Mod_ formed after lithiation, CO_2_ isotherms could not confirm the microporous structure, but the comparable BET results from Ar isotherm do not suggest any significant deviations of the two samples.

Raman spectroscopy experiments were performed to gain a more profound knowledge of the carbon structure in CuAg@C and CuAg@C_Mod_ and possible changes in the carbon structure affected by the prelithiation, as shown in Figure 1c,d. The CuAg@C Raman spectrum (Figure 1c) showed two prominent broad peaks at 1341 and 1520 cm^–1^, which the first-order Raman bands of G and D1–D4 can describe. The separation of the spectrum into five bands is based on the deconvolution method for graphitic carbon proposed by Sadezky et al.^30^ The G band reflects an ideal graphitic lattice vibration mode with *E*_2*g*_ symmetry, while the D1–D4 bands (“Defect” bands) are characteristic of disordered carbon. The first peak at 1341 cm^–1^ mainly contains the D1 band, assigned to a disordered graphitic lattice vibration mode with *A*_1*g*_ symmetry.^31^ The second peak at around 1520 cm^–1^ contains the G and D2 bands originating from a graphitic lattice vibration mode with *E*_2*g*_ symmetry. The D2 band involves vibrations of surface graphene layers, which are not directly sandwiched between two other graphene layers and are part of the disordered carbon structure.^32^ Furthermore, the shoulders at 1447 and 1195 cm^–1^ are attributed to the D3 and D4 bands, respectively, and were assigned to amorphous carbon and oxygen and nitrogen-containing carbon groups,^33,34^ which were not removed during the carbonization. The low degree of graphitization of CuAg@C results from the low carbonization temperature of 500 °C applied during the sample preparation.

In comparison to CuAg@C, the measurement data of the CuAg@C_Mod_ Raman spectrum (Figure 1d) shows an additional peak at around 1446 cm^–1^, which can be assigned to the D3 band, thereby indicating a higher degree of amorphous carbon, including formed heterogroups containing C, N, and O species after the prelithiation. By comparison of the surface area ratios of *A*_D3+D4_/*A*_total_, a measure for the surface-related disorder after the prelithiation is received (Table S1). The increase in *A*_D3+D4_/*A*_total_ from 0.07 for CuAg@C to 0.17 for CuAg@C_Mod_ indicates a significantly increased amount of amorphous carbon in the sample after the *n*-BuLi treatment. However, by calculating the *A*_D1_/*A*_G_ and *A*_D2_/*A*_G_ ratios of the samples, a decrease in the ratios is visible for CuAg@C_Mod_, indicating a higher degree of ordered carbon for the modified sample. These results can be explained by the simultaneous occurrence of two reactions during the *n*-BuLi treatment. On the one hand, the additional heat treatment at 300 °C in the glovebox causes the release of heteroatoms in the form of H_2_O, CO, and CO_2_ from CuAg@C_Mod_. Thus, the formation of aromatic carbon leads to a generally more ordered carbon structure.^35^ On the other hand, the decomposition reaction of *n*-BuLi to butene and LiH and the further reaction of LiH with butene at elevated temperatures results in more amorphous carbon at the surface for CuAg@C_Mod_.^36^

Direct current (DC) polarization measurements confirmed the change in the carbon structure was obtained toward a more ordered carbon assembly for CuAg@C_Mod_. Although resistances obtained from DC polarization are approximated values due to the high porosity of the sample, a significant deviation of the resistances around four hundred times between CuAg@C (462 MΩ) and CuAg@C_Mod_ (1.54 MΩ) can be seen in Figure S4. The increase in aromatic carbon compounds with delocalized π-electrons creates more electron pathways and thus reduces the electrical resistance.^37^

Morphological features of CuAg@C and CuAg@C_Mod_ were characterized by scanning electron microscopy (SEM), as presented in Figure 1e,f. Both samples show carbon fibers along different orientations, creating a 3D porous matrix. The diameter of the carbon fibers is uniform, about 600 nm in both cases, and large interspaces between the fibers indicate that the porous skeleton could offer a sufficient free volume for Li deposition. Scanning electron microscopy coupled with energy-dispersive X-ray spectroscopy (SEM-EDS) maps of CuAg@C were recorded to identify the long-range element distribution. Figure S5 illustrates uniform distributions of Cu, Ag, carbon (C), nitrogen (N), and oxygen (O) all over the sample. The O was derived from the oxidative stabilization step at 250 °C in the atmosphere, which is simultaneously accompanied by the formation of the oxygen-containing functional groups of carbonyls (C=O), anhydrides (O=C–O), and ether (C-OR).^38^ The doped N originates from the PAN and remains in the carbon fiber structure as a heterocyclic compounds. Carbonization temperatures far above 500 °C would be needed to remove N and O from the structure of CuAg@C while resulting in unwanted highly graphitic carbon structures.^38^

**Figure 2:**
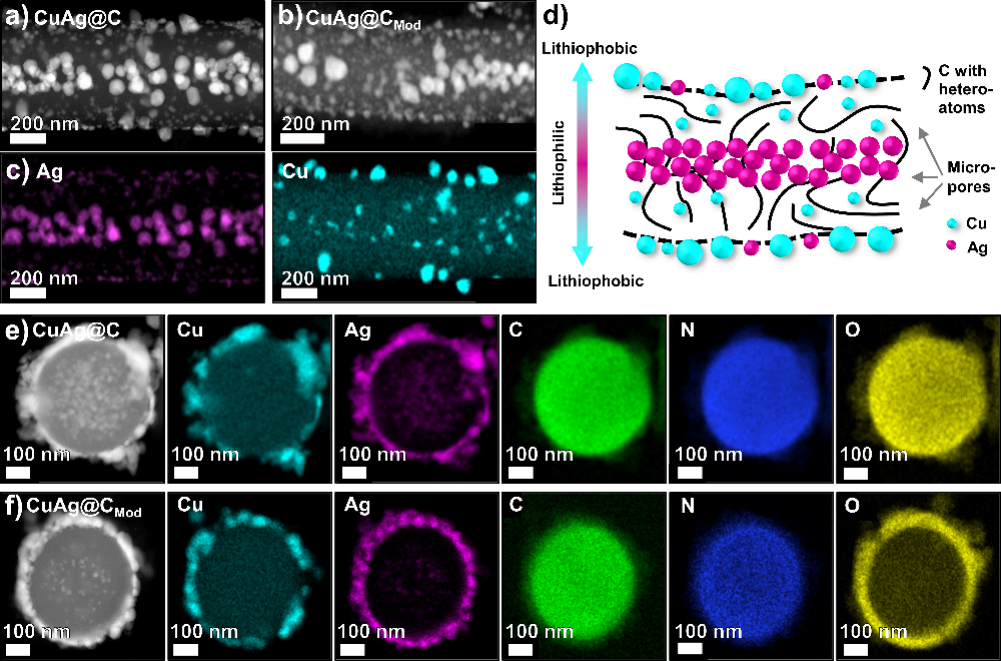
(a) STEM images of a single CuAg@C and (b) CuAg@C_Mod_ fiber. (c) STEM-EDS maps of the elemental distribution of Ag and Cu from CuAg@C. (d) Schematic illustration of the lithiophilic–lithiophobic gradient across a single fiber. (e) Cross-section STEM-EDS maps of CuAg@C and CuAg@C_Mod_ showing the field of view and element distributions of Cu, Ag, C, N, and O.

As the excitation volume of SEM-EDS was relatively large and the spatial resolution was limited, scanning transmission electron microscopy (STEM) and relevant scanning transmission electron microscopy coupled with energy-dispersive X-ray spectroscopy (STEM-EDS) mapping were performed to distinguish the distribution of the elements on a single fiber more clearly. The STEM dark field images (Figure 2a and Figure 2b) illustrate evenly distributed bright contrast spots, which are metal particles, in the center and on the surface of CuAg@C and CuAg@C_Mod_, indicating the same particle arrangement of Cu and Ag in both samples. Even if the metal particles are mainly homogeneously distributed in the center, small areas without particles can be observed and are well visible on the CuAg@C_Mod_ image. The elemental STEM-EDS maps of Ag and Cu are shown in Figure 2c and display that lithiophilic Ag particles (∼80 nm) are mainly located in the center of CuAg@C, while some significantly smaller particles are observed at the surface. Additional STEM-EDS maps of CuAg@C, showing a field of view and the elemental distribution of C, N, and O can be found in Figure S6. In contrast, the lithiophobic Cu particles with an average size of 50–100 nm are mainly distributed at the surface, and some smaller Cu particles are located in the fiber, creating a lithiophilic–lithiophobic gradient from the core to the surface along the fiber cross-section.

The arrangement of the particles can be explained by different precipitation processes of the metallic precursors (copper acetate (CuAc) and silver nitrate (AgNO_3_)) mixed with PAN in *N*,*N*-dimethylformamide (DMF) during the preparation of the electrospinning solution. While CuAc is dissociated in the solution, Ag particles are already formed due to the reduction of DMF.^39,40^ As He et al. suggested, polymer-capping of the Ag particles occurs in the solution and affects the alignment of silver particles if a high voltage is applied.^41^ Due to the static force, these align parallel to the electric field, arranging the Ag particles in a linear chain-like structure inside the electrospun polymer fibers.^41^ However, the slow reduction rate of Ag^+^ by DMF at room temperature limits the silver particle precipitation and causes small amounts of AgNO_3_ to be present and dissolved in the solution. The decomposition of CuAc, as well as the evaporation of residual nitrates, occurs in the following oxidation step at 250 °C in air and calcination step at 500 °C under Ar/H_2_ flow, releasing gases of CO_2_, NO_2_, NO, O_2_, and acetone.^42^ Besides the evaporation of the organic components, Cu and Ag particles are formed after calcination at 500 °C under a reducing atmosphere, as confirmed by the XRD results in Figure 1b.

The schematic illustration in Figure 2d visualizes the formed lithiophilic Cu and lithiophobic Ag gradients along the fiber cross-section. Theoretically, the lithiophobic Cu particles on the fiber surface avoid fast Li top-growth deposition, and the lithiophilic Ag particles inside each carbon fiber will attract the Li-ions to be deposited there, hence eliminating concentrated Li deposition.^43^ Better visualization of the lithiophilic–lithiophobic gradient in a single CuAg@C fiber can be observed in the 3D reconstruction video conducted by using X-ray tomography (Video S1). To investigate the surface element distribution after prelithiation, cross-section STEM-EDS were performed for CuAg@C and CuAg@C_Mod_, as shown in Figure 2e,f. The Cu, Ag, C, and N element distributions remained the same for both samples. The absence of the central Ag particle can be explained by sample preparation in which the carbon fibers are broken at a point without Ag particles in the fiber center. In contrast, the O maps indicate an apparent rearrangement of O after *n*-BuLi treatment. The oxygen is highly concentrated at the surface of CuAg@C_Mod_ instead of uniformly distributed in the fiber as for CuAg@C. Thus, these results graphically present the structural changes caused by the prelithiation and are consistent with the findings from the Raman analyses. The removal of oxygen atoms from the internal volume of CuAg@C_Mod_ is attributed to the additional heating step during prelithiation, while the surface accumulation of oxygen atoms is a consequence of the formed oxygen-containing species at the surface.

**Figure 3:**
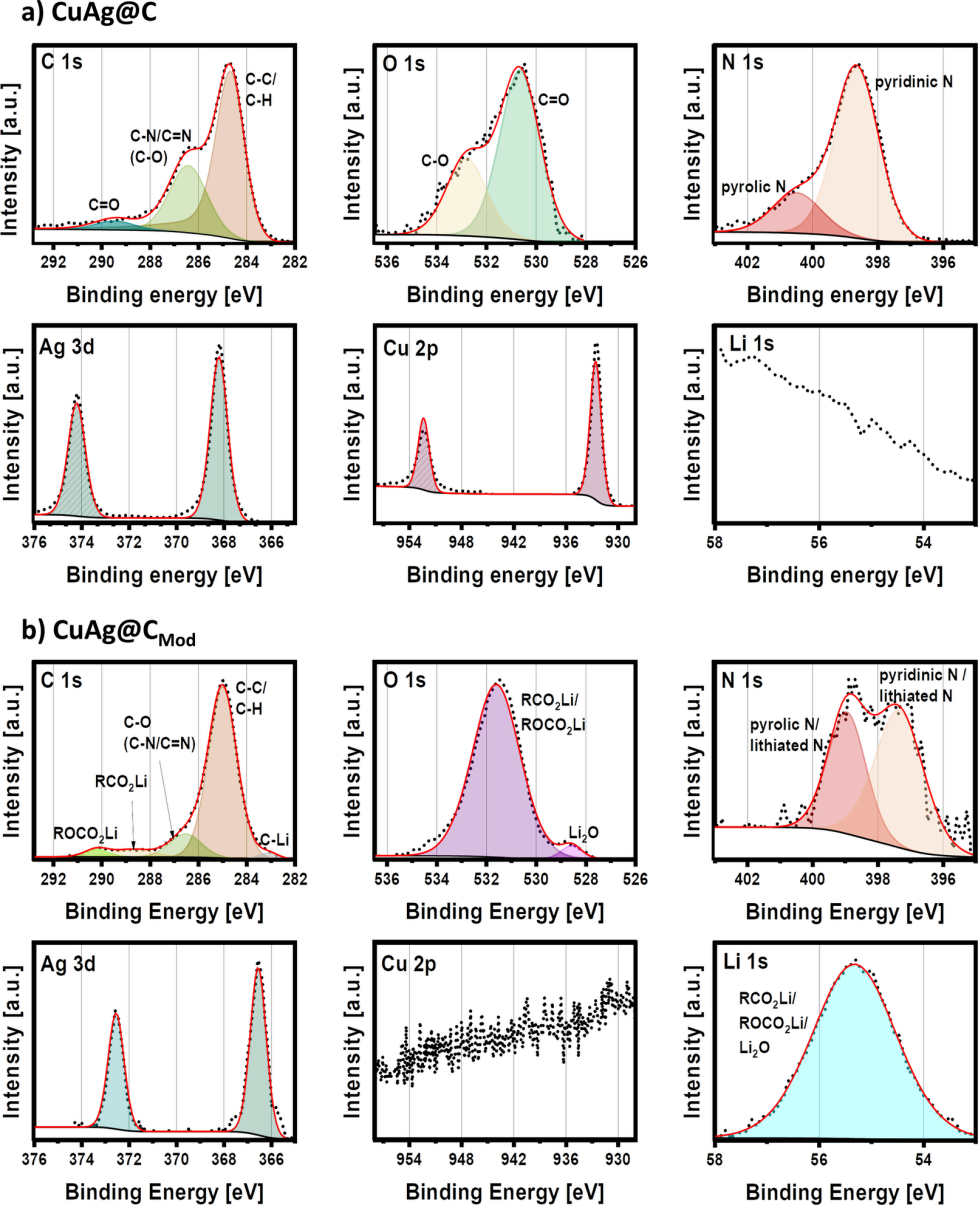
C 1s, O 1s, N 1s, Ag 3d, Cu 2p, and Li 1s XPS spectra of (a) CuAg@C and (b) CuAg@C_Mod_. The measurement parameters can be found in Table S2. All spectra are normalized, with the highest signal in each spectrum set to 1. Due to the normalization, the measurement of Li 1s for CuAg@C and Cu 2p for CuAg@C_Mod_ shows a high background noise. For comparison, the Ag 3d_5/2_ signal is set to 368.2 eV and used as the reference for CuAg@C, while for CuAg@C_Mod_, the C–C/C–H signal in the C 1s spectrum is set to 285.0 eV.

To identify the surface chemistry of CuAg@C and CuAg@C_Mod_, we performed X-ray photoelectron spectroscopy (XPS) measurements were performed. The resulting C 1s, O 1s, Ag 3d, N 1s, Li 1s, and Cu 2p spectra are displayed in Figure 3, and the measurement parameters (Table S2) and survey spectra (Figure S7) are presented in the Supporting Information. Because of the lack of a suitable common component for referencing the XPS spectra, CuAg@C spectra were referenced to the Ag 3d_5/2_ signal (Figure 3a), while the spectra of the prelithiated CuAg@C_Mod_ was referenced to the carbon in C–C/C–H at 285.0 eV in the C 1s spectrum (Figure 3b). This is due to an apparent alloy formation of Ag particles with Li during the prelithiation treatment, which shifts the spectrum toward lower binding energy. Referencing the Cu signal was not possible, as in the pretreated sample, the signal of the Cu particles was too weak and was presumably outside the probing volume of the in-house XPS.

As shown in the C 1s spectrum of CuAg@C, alongside carbon in the C–C/C–H configuration at 284.7 eV, a second signal at 286.4 eV can be observed in the C 1s spectrum. The C–C/C–H carbon can be attributed to the carbocyclic compounds in CuAg@C, while the second carbon signal indicates carbon in a heteroatomic (i.e., C=N/C—O/C—N) environment. Considering the atomic ratio (Tables S3–S12), this signal can be mainly assigned to the nitrogen-containing components. The third signal at 289.5 eV indicates the presence of C=O components on the surface. Nevertheless, the presence of C=N, C—N, C—O, and C=O can be explained by the fact that CuAg@C was treated with a low carbonization temperature of 500 °C, and therefore, residual heteroatoms of oxygen and nitrogen are still present in the material.^34^

Compared with the result of CuAg@C, significant differences are observed in the C 1s spectrum of CuAg@C_Mod_. In addition to the pronounced signal at the binding energy of 285.0 eV that can be assigned to C–C/C–H, carbon signals at 286.5 eV corresponding to C=N/C—N/C—O are significantly less intense than those in CuAg@C. This C—O/C=N/C—N signal can mainly be attributed to a C–O component rather than nitrogen-containing components in view of the atomic ratio given in Supporting Information, which results from C–C/C–H and C–O groups on the surface generated by chemical lithiation. In addition, three additional binding energies are found at 290.2, 288.7, and 283.3 eV, respectively, corresponding to ROCO_2_Li, RCO_2_Li, and lithiated carbon species as the products of prelithiation.

O 1s spectra of CuAg@C and CuAg@C_Mod_ in Figure 3 revealed changes in the oxygen-containing groups on the sample surfaces before and after prelithiation. The O 1s signals can be assigned according to the relevant C 1s spectra. For CuAg@C, the signals at 532.8 and 530.6 eV correspond to C—O and C=O bonds, respectively. For CuAg@C_Mod_, the signal at 531.3 eV is associated with the C–O, RCO_2_Li, and ROCO_2_Li components. Another less intense signal at 528.7 eV is detected due to the oxygen in Li_2_O. Li_2_O possesses the function of enhancing the mechanical strength of the surface layer formed after chemical lithiation and is helpful for the enhancement of Li-ion diffusion.^44^ Similarly, organic compounds such as RCO_2_Li and ROCO_2_Li are expected to improve the mechanical stability of the coated surface layer due to the high ductility.^45,46^ However, another study assumes that organic components with low molecular weight alkyl groups are unstable in contact with electrolytes, thus leading to the reformation of SEI and further Li-ion consumption during battery cycling.^47^ In the case of CuAg@C_Mod_, RCO_2_Li and ROCO_2_Li are most likely bound to the carbon-heteroatom network, enhancing the Li-ion transport at the sample surface.

The N 1s spectra can further identify the nitrogen-containing components already detected in the C 1s spectra. For CuAg@C, the signal at 398.6 eV can be attributed to nitrogen in a C=N environment. Another signal at 400.5 eV can be detected and assigned to nitrogen in a C–N environment. The signals originate from the pyridinic and pyrrolic N in CuAg@C, which exhibit more noise after prelithiation, as seen in the N 1s spectrum of CuAg@C_Mod_ due to a weaker signal. Additionally, since the nitrogen-containing components are part of the carbon fiber, a shift in the binding energies is observed due to the resistance gradient between the lithiated surface layer and the fibers of CuAg@C_Mod_. For the same reason, the relative amount of C=N in CuAg@C_Mod_ decreased. A different explanation for the shift of the binding energy for signals in the N 1s spectrum could be polarization induced by the Li-ions from the prelithiation.

For CuAg@C, two distinct signals are found in the Ag 3d spectrum, one at 368.2 eV (Ag 3d_5/2_) and the other signal at 374.2 eV (Ag 3d_3/2_). The combination of these peaks is ascribed to the metallic silver. As for the comparison of the N 1s spectra, the overall Ag 3d spectrum of CuAg@C_Mod_ is shifted 1.7 eV to the lower binding energy due to prelithiation, signifying the formation of Ag–Li alloy.^48^ As presented in Figure 3b, the thickness of the coating could cover the Cu 2p signal in the carbon fibers of CuAg@C_Mod_ entirely but not the Ag 3d signal, which has lower binding energy, indicating that the lithiated surface layer is about 10 nm as for the information depth of the used instrument. In addition, no signal can be detected in the Li 1s spectrum from CuAg@C, shown in Figure 3a, due to the absence of Li compounds. In contrast, a broad signal is identified in the Li 1s spectrum of CuAg@C_Mod_, corresponding to various Li compounds on the surface of the sample. The Li 1s binding energies of these compounds are unspecific and, hence, are summarized in a single peak.

Based on the structural characterizations, the prelithiation process of soaking and heating CuAg@C in *n*-BuLi hexane solution can be described as follows. Initially, soaking the sample in *n*-BuLi (CH_3_(CH_2_)_3_Li) realizes the deprotonation of pyrrolic N, amine moieties and oxygen-containing heterogroups that are typically present in carbon fiber prepared via PAN-based electrospinning, forming reaction products of carbonyl (C=O) and ester (R-CO-OR′) groups.^36^ Meanwhile, Li-ions diffused to the disordered carbon structure and associated microporosity via a redox-potential matched chemical lithiation reaction.^49^ The subsequent heating in the glovebox of the *n*-BuLi chemical lithiated sample triggers further reactions to form the above-mentioned Li compounds on the surface of CuAg@C_Mod_. While heating to 300 °C, the excess *n*-BuLi decomposed to LiH and butylene gas (CH_3_CH_2_CH=CH_2_).^36^ LiH acts as a strong reducing agent that further reacts with carbonyl/ester groups and the oxygen-containing gases of carbon monoxide (CO), CO_2_, and water (H_2_O) released from the bulk of the carbon fibers to form a complex surface comprising Li_2_O, RCO_2_Li, ROCO_2_Li, and residual LiH on the composite interlayer, with a thickness of less than 10 nm. Due to the small thickness of the modified surface and the anisotropic X-ray reflections of the carbon fiber structure, these Li compounds on the surface of CuAg@C_Mod_ were not detected by XRD. In addition to the enhancements of Li-ion transport and mechanical stability by Li_2_O, RCO_2_Li, and ROCO_2_Li, residual LiH surrounding Ag particles in CuAg@C_Mod_ could further promote and stabilize the Li plating/stripping process of CuAg@C_Mod_. Literature reported that because of the low electronegativity of H in LiH, the interfaces between LiH with high Li^+^ conductivity and Ag particles result in the formation of numerous stable built-in electric fields, which effectively boosts Li-ion diffusion from the Cu-containing lithiophobic surface of carbon fibers toward the lithiophilic Ag in the center of the carbon fibers, resulting in favorable uniform AgLi alloying/dealloying as the Ag particles are homogeneously distributed in the CuAg@C_Mod_.^50^ It is worth highlighting that the components, like ROCO_2_Li and Li_2_O, formed on the surface by soaking and heating the sample in *n*-BuLi are similar to typical SEI components formed on the anode surface after initial cycles with carbonate electrolytes in Li-ion batteries.^51^

The Li plating/stripping behavior of the prepared composite interlayers is investigated in batteries with metallic Li as counter and reference electrodes, of which the Li-ion inventory is nearly infinite. The binder-free CuAg@C or CuAg@C_Mod_ is used as an interlayer on a planar Cu foil current collector. For cycling, the initial plating was carried out at a small current of −0.1 mA cm^–2^ for 25 h and then stripped to 0.2 V at 0.1 mA cm^–2^ without a time limit to completely strip the deposited Li. The current density for subsequent plating/stripping cycles was increased to 0.5 mA cm^–2^ with time-controlled plating (5 h) and voltage-controlled stripping up to 0.2 V. Such cycling protocol can determine the stability limits of the samples in a relatively short overall time scale compared with using a short time-limit of 1 or 2 h, utilizing the same current density for plating/stripping.

**Figure 4:**
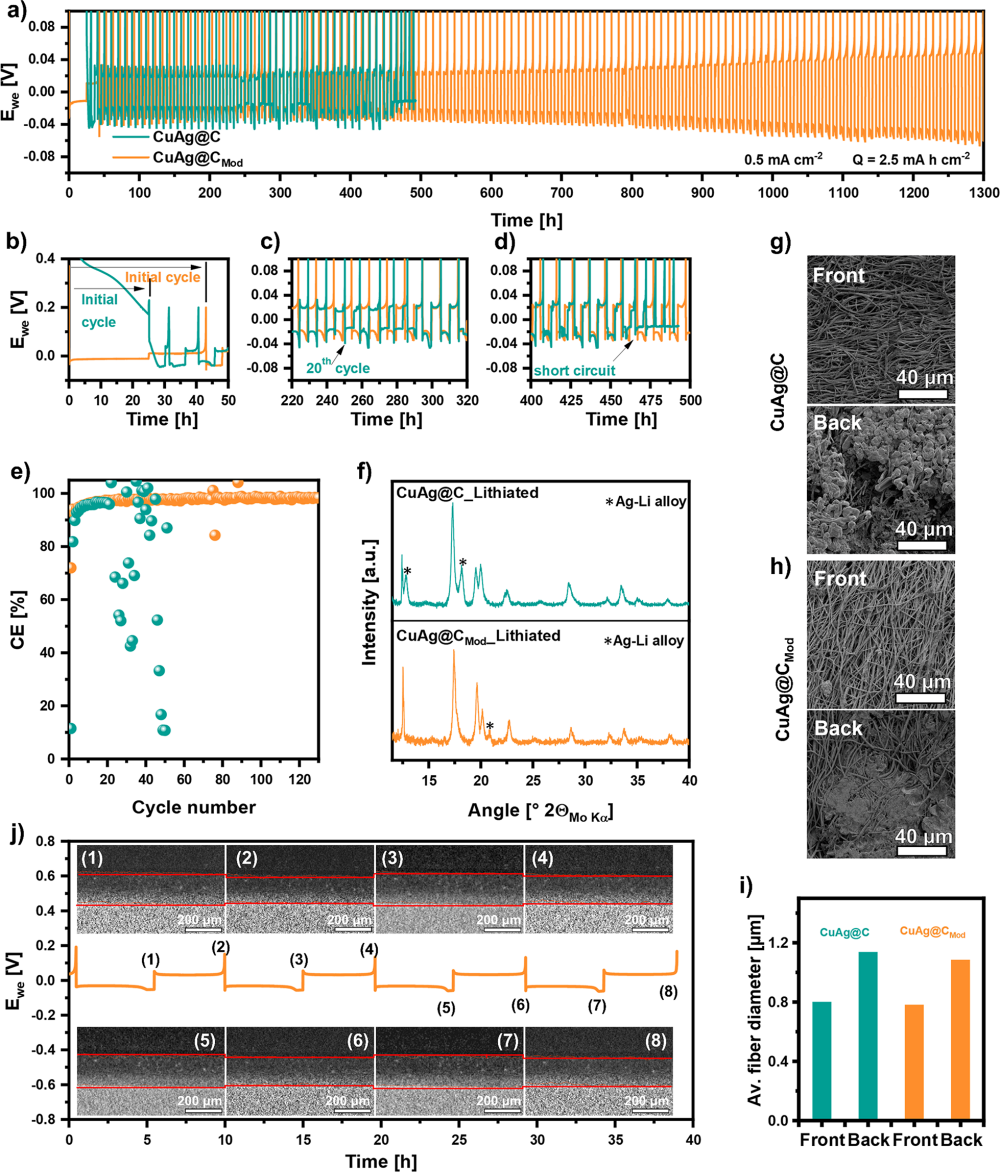
(a) Voltage profiles during Li plating/stripping and (b–d) zoomed in sections of the voltage profiles. (e) Relevant Coulombic efficiency of CuAg@C and CuAg@C_Mod_ tested in Cu foil/sample∥electrolyte∥Li cell. The initial plating was carried out at −0.1 mA cm^–2^ for 25 h and then stripped to 0.2 V at 0.1 mA cm^–2^ without a time limit. The current density for subsequent plating/stripping cycles is 0.5 mA cm^–2^ with time-controlled plating (5 h) and voltage-controlled stripping up to 0.2 V. (f) XRD pattern of lithiated CuAg@C (top) and lithiated CuAg@C_Mod_ (bottom) state after five cycles measured with Mo–Kα radiation in reflection mode. SEM images of (g) CuAg@C and (h) CuAg@C_Mod_ after 10 cycles at a Li plating capacity of 2.5 mA h cm^–2^. Pictures labeled with “Front” describe the side of the sample facing the separator and with “Back” the side facing the Cu foil. (i) Variation of the average fiber diameter of the samples after lithiation on the side facing the separator (Front) and Cu foil (Back). (j) *In situ* X-ray computed tomography images and relevant voltage profile during Li plating/stripping of CuAg@C_Mod_. The current density is 0.5 mA cm^–2^ with 5 h of time-controlled plating and voltage-controlled stripping up to 0.2 V.

The plating/stripping behaviors of CuAg@C and CuAg@C_Mod_ are depicted in Figure 4a with zoomed in versions presented in Figure 4b–d for the relevant steps. As demonstrated in Figure 4b, the formation potential of CuAg@C drops slowly during initial Li plating and reaches about 0.18 V after 25 h. The positive potential of CuAg@C after initial Li deposition results from side reactions between the carbonate electrolyte and the carbon material, including the SEI formation,^52^ Li-ion occupancy and interaction with the micropores and the N, O heteroatom-containing groups, such as irreversible Li bonding to pyridinic N compounds.^53^ The Li deposition for the cell with CuAg@C initiated after about 30 h. In contrast, when a negative current is applied, a direct drop in the potential to negative values can be observed for the cell consisting of CuAg@C_Mod_, corresponding to a direct plating of Li. During initial stripping, the potential of CuAg@C quickly reaches the 0.2 V limit, signifying the slow reaction kinetics and irreversibility of the reactions in the plating process, resulting in a low CE of about 11% (Figure 4e). The initial stripping of CuAg@C_Mod_ lasted for 18 h, corresponding to an initial CE of 72%, indicating side reactions in the initial cycle in the specific voltage range, most likely due to the irreversible Li nucleation on the planar Cu foil.

Despite the cycling conditions and low initial CE, CuAg@C can be stably cycled up to 20 times in subsequent Li plating/stripping at a high capacity of 2.5 mA h cm^–2^, as shown in Figure 4a. The relevant CE increased stepwise and reached 95.5% after seven cycles (Figure 4e). Afterward, Li dendrites grow rapidly and continuously, accompanied by randomly distributed CE values starting from the 21st cycle, causing battery failure at about 460 h. In sharp contrast, CuAg@C_Mod_ was stably cycled for 1300 h at 2.5 mA h cm^–2^ without perceivable performance fading. After several cycles, its CE reached 98% and stabilized above for the rest of the cycles. Both samples show a Ag–Li alloying process, which can be seen in the XRD pattern in Figure 4f and confirms the function of Ag particles to regulate the Li-ion distribution by its lithiophilic nature. Two additional reflections can be observed for CuAg@C_Lithiated at 12.84° and 18.13°, indicating the formation of an Ag–Li alloy. For CuAg@C_Mod__Lithiated, an Ag–Li reflex at 20.83° and a shoulder around 18.13° are observed, signifying the construction of a different Ag–Li ratio alloy compared to CuAg@C. One reason for the different lithiation state of the silver is the chemical lithiation of CuAg@C_Mod_, where the Ag is already partially alloyed with Li, as discussed in the XPS measurements.

To identify the preferred Li deposition location in the cells, CuAg@C and CuAg@C_Mod_ were investigated by SEM on the sides facing the separator (*cf*. front) and Cu foil (cf. back) after Li plating. As indicated by the results in Figure 4g and Figure 4h, Li prefers to deposit on the back side of the interlayer in both cases for two reasons. Because of the high electric resistance of CuAg@C and CuAg@C_Mod_, an electron concentration gradient from the planar Cu foil current collector toward the composite interlayer is generated while cycling. Li nucleation and deposition initially occur at the interface of the Cu foil and interlayer due to fast charge transfer reactions. Meanwhile, Ag particles on the interlayer in direct contact with the Cu foil will alloy with Li-ions. At this stage, the overall dominant process is Li deposition on a Cu current collector instead of Ag–Li alloy as most Ag particles are in the high resistive composite interlayer. While the plating of Li is continued, the deposited Li constantly grows and fills the open spaces between the Cu current collector and interlayer, touching and electrochemically “activating” more and more composite fibers in the interlayer. As the feature of lithiophilic and lithiophobic gradient from core to shell of the composite fibers, Li-ions could easily reach the big Ag particles in the fiber center, resulting in homogeneous expansion of the fibers. As shown in Figure 4i, the expansion of the fibers on the “back” of the samples is similar for CuAg@C and CuAg@C_Mod_ (1137 to 1084 nm). Compared to the pristine fiber diameter before cycling (∼600 nm) shown in Figure 1e,f, the slight expansion of the fibers in the “front” is mostly due to electrolyte uptake. Despite no metallic Li detected in the “front” of both samples (Figure 4g,h), significantly less concentrated Li plating is observed on the “back” of CuAg@C_Mod_ compared with that of CuAg@C due to the substantially lower resistivity of CuAg@C_Mod_ where more fibers are activated for Li deposition.

The homogeneity of Li deposition in the case of CuAg@C_Mod_ is proven by *in situ* X-ray computed tomography (CT) using a standard perfluoro alkoxy alkane Swagelok-type cell case (Figure S8). Figure 4j shows the galvanostatic cycling curve of CuAg@C_Mod_ during *in situ* CT measurements with the corresponding images recorded at each Li plating/stripping step. Due to the high standard deviation of ±8 or 9 μm and the suboptimal resolution of the images, the expansion should only be qualitatively related to a constant volume change. The postdischarging images indicate the absence of concentrated Li growth since a consistent volume expansion (20 μm) of the CuAg@C_Mod_ interlayer during each plating/stripping cycle is obtained (Table S13). This verifies uniform Li deposition at the bottom and within the interlayer, underscoring the advantages of the stress-minimizing fiber structure of CuAg@C_Mod_.

**Figure 5:**
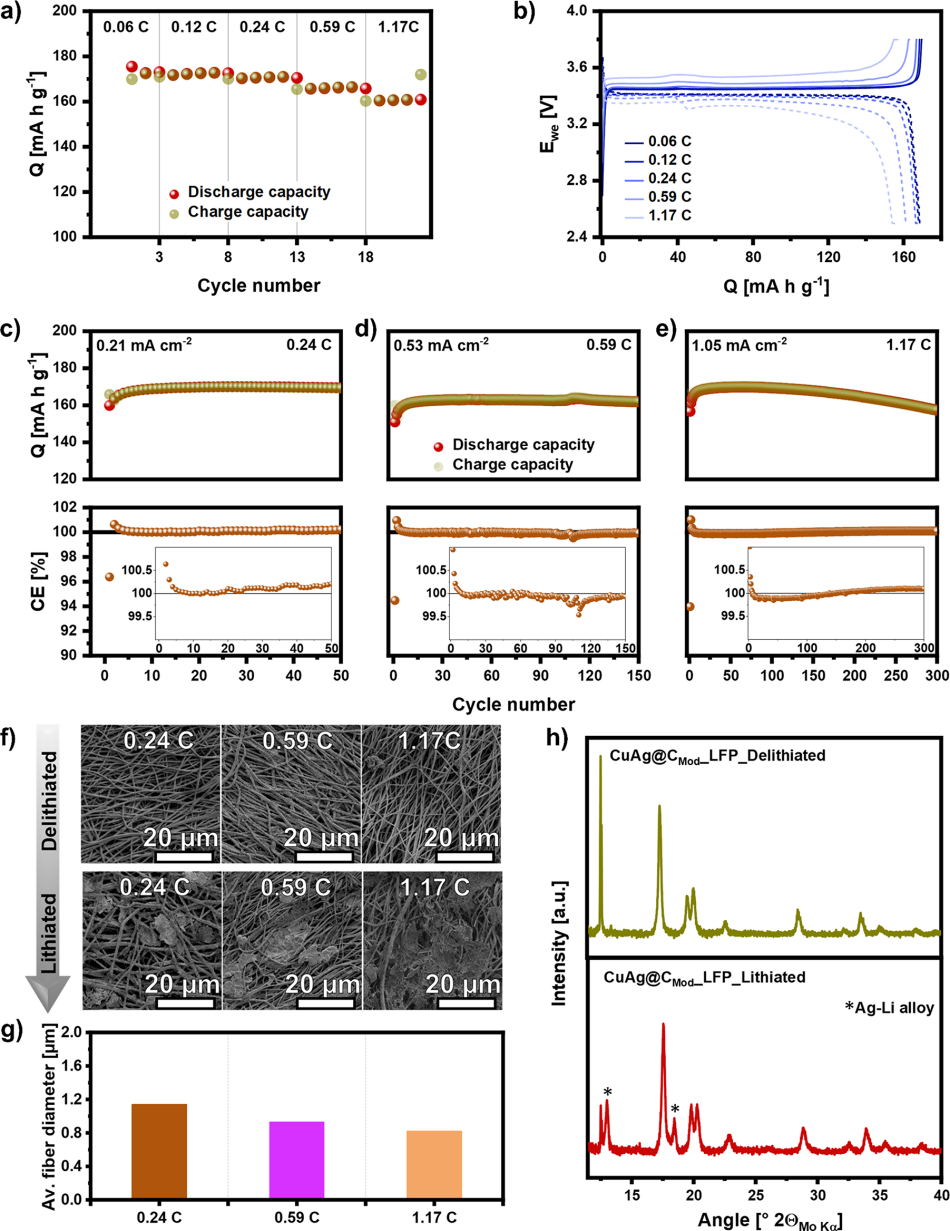
(a) Rate performance and (b) corresponding charge–discharge curves of the tested in Cu foil/CuAg@C_Mod_∥electrolyte∥LFP cells cycled at 0.06, 0.12, 0.24, 0.59, and 1.17 C, respectively, at 25 °C. (c–e) Long-term cycling and relevant CE of the cells cycled at 0.24, 059, and 1.17 C, respectively. (f) SEM images of CuAg@C_Mod_ in lithiated and delithiated states after five cycles at 0.24, 0.59, and 1.17 C and (g) the corresponding average diameters of the fibers facing Cu current collector after lithiation. (h) XRD pattern of CuAg@C_Mod_ in lithiated and delithiated states after five cycles at 1.17 C measured with Mo–Kα radiation in the reflection mode.

To evaluate the electrochemical performance of CuAg@C and CuAg@C_Mod_ current collector interlayers in zero-excess Li metal batteries with limited Li-ion inventory, commercial LFP sheets with an active material loading of 5 mg cm^–2^ were used as the cathode for battery assembly. Similar to the initially long stabilization process for Li plating/stripping measurement, the potential of the battery with CuAg@C interlayer does not increase even with 400 h of charging under a small current density of 0.06 C (Figure S9). This is because the limited Li-ion inventory in the zero-excess Li metal battery cannot afford the Li-ion consumptions for SEI formation and side reactions of Li with defects in carbon fibers. In contrast, zero-excess Li metal batteries using CuAg@C_Mod_ as current collector interlayer can be stably cycled at different current densities of 0.06, 0.12, 0.24, 0.59, and 1.17 C, with high specific discharge capacities of 172.3, 172.6, 171.0, 166.5, and 161.0 mA h g^–1^, respectively, as shown in Figure 5a. Even at a high C-rate of 1.17 C, about 92% of the theoretical capacity of LFP is achieved. Charge–discharge curves of the cell cycled at different C-rates are presented in Figure 5b, reflecting the LiFePO_4_ ↔ FePO_4_ redox reaction with small overpotentials while cycling at different current densities.

One of the advantages of prelithiated electrodes is that no formation cycles using small currents are necessary for battery cycling as the preformed stable SEI and prelithiation can compensate for initial Li-ion loss from electrolyte-electrode decomposition reactions up to a certain extent.^54,55^ Therefore, the zero-excess Li metal batteries with CuAg@C_Mod_ interlayer were directly tested at different current densities of 0.24, 0.59, and 1.17 C, respectively, to investigate the long-term cycling stability. The cycling performance and corresponding initial and end cycling curves are presented in Figure 5c–e and Figure S10, respectively. Notably, the specific capacity of cells tested at three different C-rates shows a rising trend in the first six cycles and stabilizes in the following charge–discharges. A closer look at the CE over the cycles helps to reveal the electrochemical processes of the cells. As shown in Figure 5c–e, the initial CE of the cells is about 95%, as typically for LFP cathodes reported in the literature, caused by the crystal structure rearrangement of LFP after initial delithiation.^56,57^ Unlike Li batteries using metallic Li as the anode in which the CE remains below but close to 100% after the first cycle, the zero-excess Li metal batteries with CuAg@C_Mod_ interlayer exhibited a CE of approximately 101% for the second cycle and stepwise decreased to almost 100% for the sixth cycle. This effect can be explained by an increased electrolyte penetration of the LFP cathode, achieved by initial cracking of the carbon, in combination with a redistribution of the stored Li-ion after the prelithiation of CuAg@_Mod_.^58,59^ Thereby, additional Li-ions can be extracted from CuAg@C_Mod_ and stored or consumed in the cathode to create a stable cathode–electrolyte interphase during these cycles.^60^ After the sixth cycle, the CEs of all the cells are high and varied in a small range of 99.9–100.1%, indicating occasional compensation from the surface components of CuAg@C_Mod_ for the Li-ion loss caused by side reactions in the system, thus guaranteeing long-term cycling stability.

Because of the increase of the discharge capacity in the initial several cycles, the capacity retentions given in the following are compared to the maximum discharge capacity among the cycles instead of the first cycle. For the cells cycled 50 times at 0.24 and 150 times at 0.59 C, almost no capacity fading is observed over the cycles. The discharge capacity retentions are 99.7% and 99.3% after 50 and 150 cycles, respectively, corresponding to 99.5% and 95.3% of the theoretical capacity of the LFP cathode. For the cell cycle at a high C-rate of 1.17 C, the specific discharge capacity of the battery after 300 cycles is 157.4 mAh g^–1^, corresponding to 92.6% retention of the maximum capacity. The long cycling stability indicates the highly reversible Li-ion shuttle process through the components in the system with the CuAg@C_Mod_ interlayer.

The morphologies of CuAg@C_Mod_ facing Cu foil at lithiated and delithiated states after five cycles in the batteries at C-rates of 0.24, 0.59, and 1.17 C, respectively, were recorded by SEM. As demonstrated in Figure 5f, in accordance with the excellent capacity retentions and the high CE of the batteries, no metallic Li residues can be seen on CuAg@C_Mod_ samples after delithiation at any of the tested current densities. Different morphologies of the lithiated CuAg@C_Mod_ samples cycled at different currents are observed. With increasing the cycling currents from 0.24 to 1.17 C, more metallic Li is found in the pores of the interlayers, accompanied by the stepwise decreased average fiber diameter from 1142 at 0.24 C to 931 and 822 nm at 0.59 and 1.17 C, respectively (Figure 5g). These results reveal the different reaction dynamics of Li-ions deposited at the copper/CuAg@C_Mod_ interface and directly interacting with the interlayer under different electric fields. The interaction between Li-ions and CuAg@C_Mod_ includes not only direct Li deposition on the interlayer but also Ag–Li alloying, as evidenced by the XRD results shown in Figure 5h. Even at 1.17 C, reversible Ag–Li alloying is identified. On the other hand, under stronger electric fields, Li-ions are driven by greater force to continuously deposit on the already nucleated Li at the Cu/CuAg@C_Mod_ interface due to the different Li chemical potentials of metallic Li and CuAg@C_Mod_. While benefiting from the limited Li-ion inventory in the system and the large interspaces of the interlayer, the battery with the CuAg@C_Mod_ interlayer was stably cycled over 300 times at 1.17 C with negligible capacity fading.

**Figure 6:**
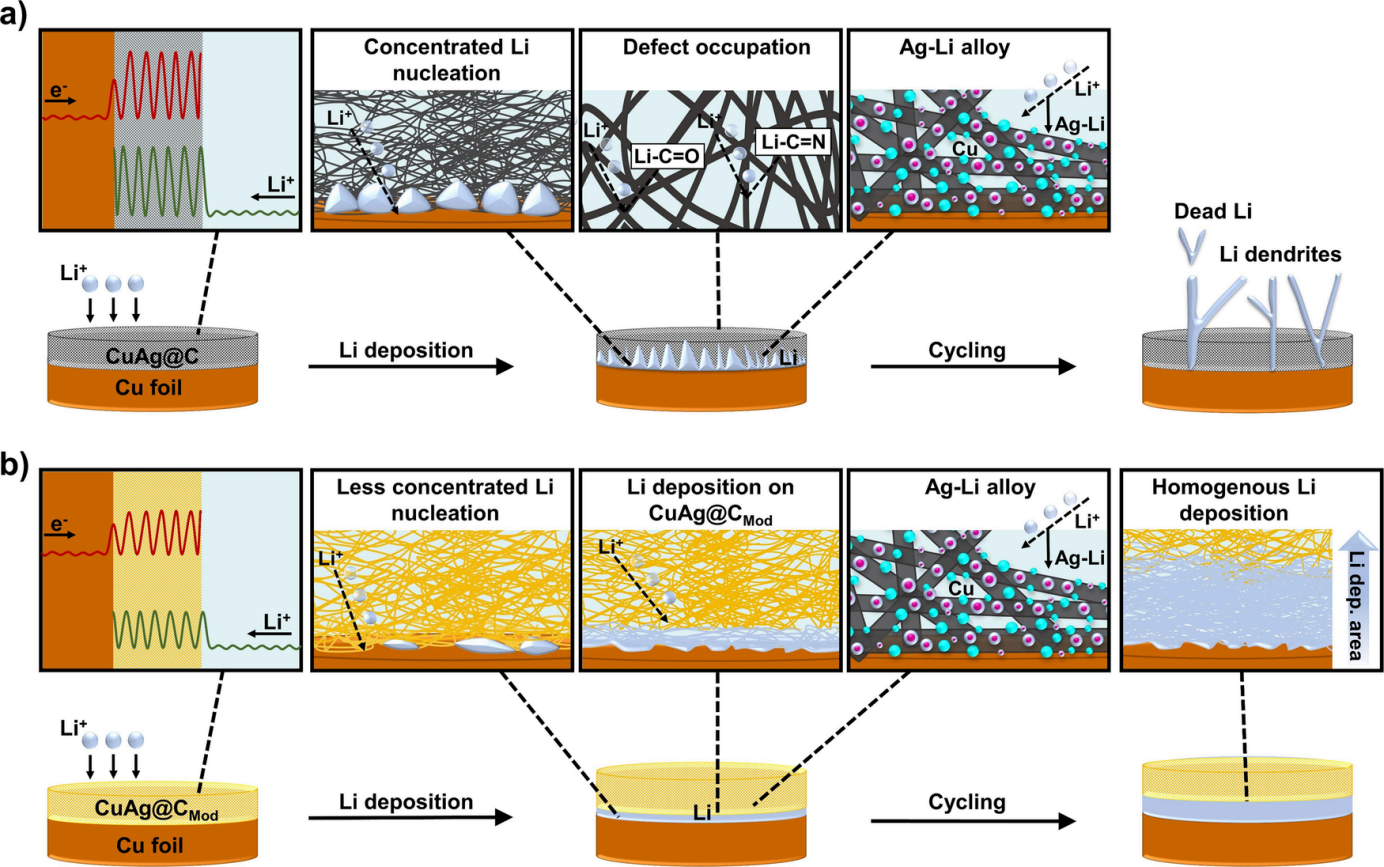
Schematic illustration of the Li deposition/dissolution behaviors of CuAg@C (a) and the chemical prelithiated CuAg@C_Mod_ (b).

Combined with the above Results and Discussion, Figure 6 schematically illustrates Li deposition processes in the cells with CuAg@C and CuAg@C_Mod_, respectively. As shown in Figure 6a, CuAg@C without chemical prelithiation, characterized by a highly amorphous carbon structure, possesses high energy barriers for electron and Li-ion migration. While Li deposition, due to the significantly lower electronic resistance of the Cu foil compared with CuAg@C, Li-ions are initially nucleated on the Cu surface and continuously deposited there through the liquid electrolyte. Meanwhile, excessive Li-ions are consumed, especially at the initial cycles, for the occupancy of defects in the carbon matrix and forming SEI on the large surface area of the sample. Despite the highly reversible Ag–Li alloy process, this mainly happens at the Ag particles on the surfaces of carbon fibers in direct contact with Cu foil due to reduced electronic resistance. Most Ag particles in the carbon fibers are hardly reachable by the Li-ions due to the defect occupation in the carbon matrix. The dominant process for CuAg@C is Li deposition on a planar lithiophobic Cu current collector so that nonuniform Li deposition becomes more distinct while cycling. Li dendrites eventually formed, limiting the plating/stripping process to a few cycles. In zero-excess Li metal batteries, the limited Li-ion inventory cannot compensate for the Li-ion loss of the side reactions. Consequently, the battery cannot be cycled.

Chemical prelithiated CuAg@C_Mod_ exhibited lower energy barriers for electron and Li-ion migration compared with CuAg@C, enabled by 10 nm thick surface Li-rich compounds (Li_2_O, RCO_2_Li, ROCO_2_Li, and LiH) originating from the redox reactions of *n*-BuLi and heteroatoms in the low-temperature calcined carbon fiber matrix as well as O releasing during the reactions. As illustrated in Figure 6b, although Li-ions can still deposit on the Cu current collector, the Li plating on CuAg@C_Mod_ and energetically preferred Ag–Li alloying process combined to contribute during cycling ensure long-term Li plating/stripping. In zero-excess Li metal batteries, the consumption of limited amounts of Li-ions can be compensated for by the thin Li-rich compounds on the surface CuAg@C_Mod_. While cycling, the formed Ag–Li alloy reduces the electrical resistance further, thus shifting the preferred Li deposition toward the interlayer instead of Cu foil. The formation of dead Li, caused by the deposited Li on the Cu foil, is hindered through the CuAg@C_Mod_ matrix, which could wrap and connect the deposited Li with the composite fibers in the interlayer containing lithiophilic Ag. The negative Gibbs energy of the Ag–Li alloy affects a preferential formation of the alloy, whereby thermal decomposition due to concentrated charge accumulation is eliminated. These effects synergistically increase the cycling life compared to other zero-excess Li metal batteries reported in the literature (Table S14).

## Conclusion

For zero-excess Li metal batteries or, e.g., anode-free batteries, Li is continuously consumed to build a stable interface between the Cu current collector and electrolyte, resulting in a substantial capacity loss in the initial cell activation and capacity decay while cycling, which hinders their practical application. Our findings demonstrated an effective approach to realizing long lifespan zero-excess Li metal batteries without a formation cycle using a 3D lithiophilic/-phobic interlayer with a Li-rich surface modification. The advantages of using the composite interlayer in a zero-excess Li metal battery design are (i) the porous structure reduces built-in stress during Li deposition/dissolution and the interconnected fibers mechanically and thermodynamically protect the deposited Li on the current collector, (ii) the formation of an Ag–Li alloy induces uniform Li-ion deposition, and (iii) the chemical prelithiation induced a thin Li-rich surface coating that can eliminate electrolyte side reactions and guaranteeing the high CE and calendar lives of zero-excess Li metal batteries. A homogeneous Li deposition is received even at high current density and without formation/activation cycles. These results demonstrate the possibility of practical zero-excess Li metal batteries via improved cell design.

## Experimental Section

### Fabrication of CuAg@C and CuAg@C_Mod_

All chemicals were used as received without pretreatment. First, 3.776 g of PAN (*M*_w_ = ∼150000) and 2.815 g of CuAc were dissolved in 40 mL of anhydrous DMF and stirred for 1 day at room temperature. Afterward, 2.633 g of AgNO_3_ was added to the solution and stirred for another day until a cyan-colored solution was obtained. Electrospinning is carried out with the prepared solution using an electrospinner with a rotating drum collector in a horizontal position (IME Medical Electrospinning, The Netherlands) under controlled climate conditions of 25 °C and 30% relative humidity. The solution was transferred to a syringe and pumped via an automatic syringe pump with a flow rate of 20 μL min^–1^ through four needles. The needles with an inner diameter of 0.8 mm were horizontally moved with a speed of 20 mm s^–1^ in ±55 mm range with a turn delay of 500 ms. The applied rotation speed for the rotating drum was 700 rpm, and the distance from the collecting drum to the needle tip was 140 mm. The electrospinning process was performed at a voltage of 25 kV for a total time of 6.5 h. The obtained green body was cross-linked in the air for 15 h at 250 °C and followed with a reduction step under Ar/H_2_ (3 vol %) flow for 2.5 h at 500 °C with a heating rate of 5 °C min^–1^ to obtain the final CuAg@C free-standing membrane. The metal content in the sample is approximately 50 wt % as identified by thermal gravimetric analysis (TGA) for CuAg@C (Figure S11). Chemical prelithiation was carried out in an Ar filled glovebox with water and oxygen contents below 0.1 ppm. CuAg@C was cut to a disk with a diameter of 11 mm and immersed in 1 mL of 2.5 M *n*-BuLi/hexane solution at room temperature for 3 days in a glass vial. Subsequently, excess *n*-BuLi solution was removed, and the saturated CuAg@C was heated to 300 °C for 30 min.

### Structural Characterizations

XRD measurements were carried out using an EMPYREAN X-ray diffractometer (PANalytical, The Netherlands) with Mo Kα radiation in transmission and reflection mode. The operation is at 45 kV with a current of 40 mA. The step size is 0.008° from 5 to 95° 2θ with a total measurement time of 58 min per sample. For transmission mode measurements, the samples were placed between nonreflective Kapton foils. The XRD measurements from cycled batteries against Li metal were taken at the lithiated state after a formation cycle and five additional plating/stripping cycles and at the lithiated/delithiated state after five cycles at 1.17 C for the cells against LFP. To protect the samples from air contamination, they were placed between magic tape (Scotch Magic) and silicon wafer substrate in the glovebox. The sample preparation results in a reflection mode measurement due to the X-ray impermeability of the silicon wafers. The reference XRD patterns are Si (ICSD 51688),^61^ Cu (ICSD 136042),^62^ and Ag (ICSD 22434).^63^ The specific surface area was determined through BET measurements by Ar and CO_2_ adsorption, and the automatic adsorption analyzer Micro 300C-02-Analysis Station (JWGB Instruments, China) was used. The reproducibility and accuracy of specific surface area results of the samples are ±0.4 m^2^ g^–1^, confirmed with different samples. Monte Carlo simulation was conducted using Quantachrome ASiQwin- Automated Gas Sorption Data software (Anton Paar, Germany). FEI Quanta FEG 650 (FEI, U.S.A.) was used for SEM analysis with an EDAX–Octane 70 mm2 EDS detector (EDAX-Ametek, U.S.A.). For air/moisture-sensitive samples, a K&W transfer module (Kammrath and Weiss, Germany) was used to transfer the samples from the glovebox directly to the SEM chamber. The module opens in the SEM chamber under vacuum. Hence, the samples are not exposed to the ambient atmosphere. An acceleration voltage of 1 kV and a spot size of 1 were used for SEM measurements to preserve the true-to-life morphology and avoid electron beam damage on the sample surface. For the corresponding SEM-EDS mappings, the acceleration voltage was increased to 10 kV and the spot size to 6. Scanning transmission electron microscopy (STEM) experiments were conducted using an FEI Titan G2 80–200 microscope with a Cs-probe corrector and a HAADF detector. The microscope was operated at 200 kV, and the probe semi angle was 24.7 mrad. Elemental maps were taken by energy-dispersive X-ray spectroscopy (EDS) using four window-less large-solid-angle symmetrical Si drift detectors. The dispersion was set, so the energy range of 0–20 keV was detected, including Cu K-lines at 8.1–8.9 keV but no Ag K-lines at 22.26 keV. For the reconstruction movie, a tilt series with a step size of 2° was recorded and the 3D images were reconstructed using a Matlab script. Raman microspectroscopic measurements were performed using a WITec alpha300R Raman microscope (OXFORD Instruments, U.K.) using a solid-state 532 nm excitation laser, 600 lines mm^–1^ grating, and a laser power of 1.95 mW. The Raman spectra were collected with a point focus lens and a 50× objective on an area of 50 μm × 50 μm with 25 points per line and 25 lines per image. Each spectrum at each point was collected with 5 s of integration time. The collected spectra were corrected for cosmic rays and averaged to obtain a representative spectrum for each sample. Furthermore, the measurements were conducted in an in-operando ECC-Opto-Std cell (EL-CELL, Germany) equipped with a borosilicate glass window to protect the samples from the air. Curve fitting for determining the spectral parameter was performed with Origin (Originlab Corporation, 2021, U.S.) and followed the deconvolution method proposed by A. Sadezky into five bands with Lorentzian or Gaussian contributions.^30^ A Voigt function was used, and the D1, D2, and D4 bands were described by considering exclusively Lorentzian contributions for D1, D2, and D4. D3 was fitted by considering exclusively Gaussian contributions. For the G band, a combination of Lorentzian and Gaussian shape lines was found to achieve better goodness of fit values and a better description of the observed spectrum. XPS was recorded on a Kα spectrometer connected to a glovebox (Thermo Fisher, U.S.A.), enabling measurements without air contamination. The instrument has an Al–Kα X-ray source and is operated at a 10–9 mbar base pressure. The elements were measured with a pass energy of 50 eV and a spot size of 400 μm on the sample. The survey was measured with a pass energy of 200 eV and the same spot size as those of the elements. The samples were evaluated by using the software Avantage (ThermoFisher). The exact measurement parameters and fit parameters can be taken from the appendix (Tables S2–S12). The homogeneity of the sample is confirmed by measurements at a second point, as the results are shown in Supporting Information (Figure S12, Tables S15–S24). The *in situ* X-ray CT measurement was performed at a ZEISS XRADIA Versa 620 with an accelerating voltage of 90 kV. The voxel size, defined by the combination of geometrical and optical (4× objective) magnification, was 2.5 × 2.5 × 2.5 μm^3^. The acquisition time for one record took 2.5 h with an exposure time of 6 s per projection. A high pass filter was used to reduce the beam hardening artifacts. A plastic Swagelok-type cell battery case shown in Figure S8 with the configuration Cu foil/sample||electrolyte||Li using the same current density, electrolyte, and separators as for the Li plating/stripping tests was utilized for the X-ray CT measurements. The images were captured during an open circuit voltage step, and the measurements began after the fifth plating cycle (formation cycle plus three cycles with a Li plating capacity of 2.5 mA h cm^–2^). The experiment was controlled by a custom script reading information from potentiostat and triggering the scans automatically if the current value remained near zero for longer than 1 min. It was achieved thanks to the exposed application programming interface of the Versa scanner and implemented similarly to the previous work.^64^ After the acquisition, images were reconstructed and segmented using thresholding and a combination of standard morphological operations, namely, dilation and erosion. Fibers’ voxels, delineated from other features, were then integrated in the normal direction to create two-dimensional thickness maps. Finally, the fiber thickness at the given stage was defined as the average value from the corresponding 2D map. TGA was performed on a NETZSCH TGA/STA-QMS 403D thermoanalyzer (Germany) between 30 and 800 °C with a heating rate of 5 °C min^–1^ under the atmosphere.

### Electrochemical Measurements

Li plating/stripping tests were carried out on the battery configuration of Cu foil/sample||electrolyte||Li in CR2032 coin cells with a polypropylene separator (Celgard 2400). The electrolyte is a dual lithium salt carbonate solution containing 0.6 M lithiumtetrafluorborat, 0.6 M lithiumdifluoro(oxalato)borate in fluoroethylene carbonate, and diethyl carbonate (1:2, vol/vol). The cells were initially Li plated for 25 h and stripped to 0.2 V at a small current density of 0.1 mA cm^–2^, and subsequently Li plated for 5 h and stripped to 0.2 V at a high current density of 0.5 mA cm^–2^. Cycling measurements of the zero-excess Li metal batteries Cu foil/sample||electrolyte||LFP were conducted in Swagelok-type batteries at different current densities in a cutoff voltage range of 2.5 to 3.8 V vs Li^+^/Li with a subsequent constant voltage step until 10% of the previously applied current was reached. LFP electrode disks (NEI Corporation, U.S.A.) with an active loading of 5 ± 0.4 mg cm^–2^ were cut into 11 mm disks and used as the cathode. The separator and electrolyte are the same as those used for the Li plating/stripping tests. The DC polarization method was chosen to determine the electronic resistance. Therefore, a symmetric cell setup containing a stainless steel disk||sample||stainless steel disk was constructed without any electrolyte. The current vs time curves were recorded at 2 V for 5 min. All the cells were tested using multichannel potentiostats (VMP3, MPG-2, BioLogic, France) at 25 °C, controlled by a climate chamber (Binder, Germany).

## Supporting Information Available

Ar adsorption isotherms of CuAg@C and CuAg@C_Mod_ and the calculated BET surface area, CO_2_ adsorption isotherms of CuAg@C, cumulative pore volume and particle size distribution of CuAg@C, spectral parameter for the first-order Raman bands of CuAg@C and CuAg@C_Mod_, DC polarization measurement of CuAg@C and CuAg@C_Mod_, SEM-EDS images of CuAg@C, STEM-EDX images of CuAg@C, measurement details for XPS scans, survey measurement of the XPS spectrum for CuAg@C and CuAg@C_Mod_, detailed fitting parameters for all recorded XPS spectra (C 1s, O 1s, N 1s, Li 1s, Ag 3d, Cu 2p) for CuAg@C and CuAg@C_Mod_, digital photograph of the used standard perfluoroalkoxy alkane Swagelok-type cell for X-ray CT measurements, calculated average thickness of CuAg@C corresponding to the *in situ* X-ray CT measurements, charge curve of CuAg@C against LFP cathode, charging–discharging curves of CuAg@C_Mod_ against LFP at 0.24, 0.59, and 1.17 C, literature overview of zero-excess Li metal battery performances, TGA curve of CuAg@C, C 1s, O 1s, N 1s, Ag 3d, Cu 2p, and Li 1s XPS spectra of CuAg@C and CuAg@C_Mod_ for a second measured point and the corresponding fitting details (PDF)
Reconstruction movie of CuAg@C (MP4)

## Abbreviations

Li_2_O: lithium oxide
RCO_2_Li: lithium carboxylate
ROCO_2_Li: lithium carbonates
LiH: lithium hydride
XRD: X-ray diffraction
BET: Brunauer–Emmett–Teller
SEM: scanning electron microscopy
SEM-EDS: scanning electron microscopy coupled with energy-dispersive X-ray spectroscopy
STEM: scanning transmission electron microscopy
STEM-EDS: scanning transmission electron microscopy coupled with energy-dispersive X-ray spectroscopy
EDS: energy-dispersive X-ray spectroscopy
CuAc: copper acetate
AgNO_3_: silver nitrate
DMF: *N*,*N*-dimethylformamide
XPS: X-ray photoelectron spectroscopy
CO: carbon monoxide
H_2_O: water
DC: direct current
CE: Coulombic efficiency
SEI: solid electrolyte interphase
*n*-BuLi: *n*-butyllithium
LFP: LiFePO_4_
PAN: polyarcylonitrile
C: carbon
N: nitrogen
O: oxygen
CT: computed tomography
TGA: thermal gravimetric analysis
