# Supplementary material for: Chemical Prelithiated 3D Lithiophilic/-Phobic Interlayer Enables Long-Term Li Plating/Stripping
Source: ACS Nano. 2024 Jun 28;18(27):17924–38. doi: 10.1021/acsnano.4c04507 (PMC11238590; doi:10.1021/acsnano.4c04507)
Supplement: Supplementary file 1 — nn4c04507_si_001.pdf [file nn4c04507_si_001.pdf]

Supporting Information

Chemical prelithiated 3D lithiophilic/-phobic interlayer enables long term Li plating/stripping

*Sandro Schöner‡[1,2], Dana Schmidt‡[1,2], Xinchang Chen[3], Krzysztof Dzieciol[1], Roland Schierholz[1], Pengfei Cao[4], Ahmad Ghamlouche[5], Fabian Jeschull[5], Anna Windmüller[1], Chih-Long Tsai[1], Xunfan Liao[6], Hans Kungl[1], Gui-Ming Zhong[3], Yiwang Chen[6], Hermann Tempel[1], Shicheng Yu[1] * and Rüdiger-A. Eichel[1,2]*

[1]S. Schöner, D. Schmidt, R. Schierholz, K. Dzieciol, A. Windmüller, C. Tsai, Hans Kungl, H. Tempel, S. Yu, R.-A. Eichel

Institute of Energy and Climate Research (IEK-9: Fundamental Electrochemistry), Forschungszentrum Jülich, 52428 Jülich, Germany
E-mail: s.yu@fz-juelich.de

[2]S. Schöner, D. Schmidt, R.-A. Eichel

Institut für Materialien und Prozesse für elektrochemische Energiespeicher und wandler, RWTH Aachen University, 52074 Aachen, Germany

[3]X. Chen, G.-M. Zhong

Laboratory of Advanced Spectro-electrochemistry and Li-ion Batteries, Dalian Institute of Chemical Physics, Chinese Academy of Sciences, Dalian 116023, China

[4]P. Cao

Ernst Ruska-Centre for Microscopy and Spectroscopy with Electrons, Forschungszentrum Jülich, 52428 Jülich, Germany

[5]A. Ghamlouche, F. Jeschull

Karlsruher Institute of Technologie (KIT), Institute for Applied Materials-Energy Storage Systems (IAM-ESS), 76344 Eggenstein Leopoldshafen, Germany

[6]X. Liao, Yiwang Chen

National Engineering Research Center for Carbohydrate Synthesis/Key Lab of Fluorine and Silicon for Energy Materials and Chemistry of Ministry of Education, Jiangxi Normal University, 330022 Nanchang, China

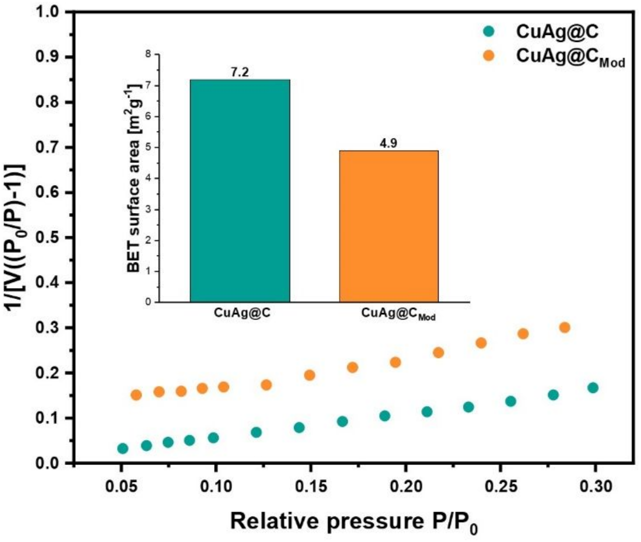

Figure S1 Ar adsorption isotherm of CuAg@C and CuAg@C_{Mod} measured at 273 K. The calculated BET surface area is 7.2 m^2 g^{-1} for CuAg@C and 4.9 m^2 g^{-1} for CuAg@C_{Mod}.

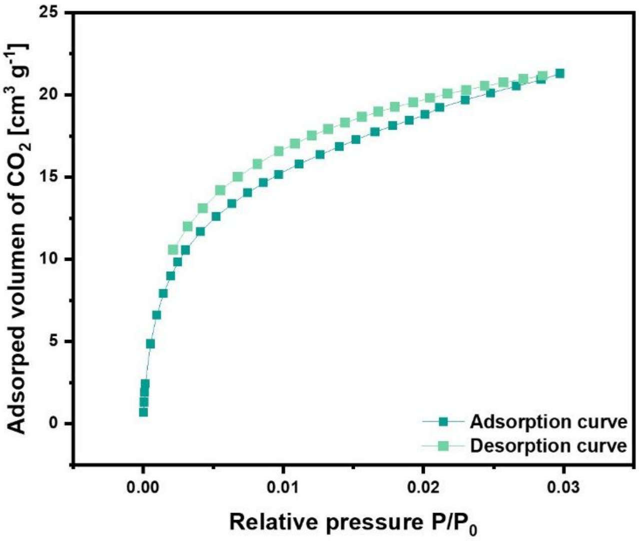

Figure S2 CO_2 adsorption isotherm of CuAg@C measured at 273 K.

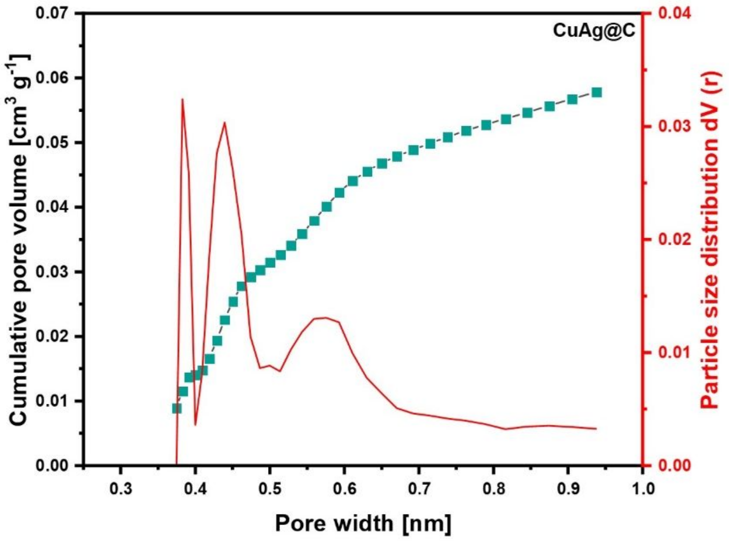

Figure S3 CO_2 adsorption properties of CuAg@C measured at 273 K, showing the cumulative pore volume and the particle size distribution obtained by Monte Carlo calculations. The average pore volume is 0.058 cm^3 g^{-1}, with a surface area of around 209.13 m^2 g^{-1} and pore width of 0.37 nm.

Table S1 Spectral parameter for the first-order Raman bands of CuAg@C and CuAg@C_{Mod}, showing the band position (Stokes Raman shift), full width at half maximum (FWHM), the peak area (A) and the peak area ratios.

Band	Parameter	CuAg@C	CuAg@C_{Mod}
G	Position [cm^{-1}]	1520	1530
	FWHM [cm^{-1}]	143	127
	A [cm^2]	7612	62574
D1	Position [cm^{-1}]	1341	1344
	FWHM [cm^{-1}]	153	195
	A [cm^2]	36355	191946
	A_{D1}/A_G	4.78	3.07
D2	Position [cm^{-1}]	1590	1595
	FWHM [cm^{-1}]	107	92
	A [cm^2]	11233	72837
	A_{D2}/A_G	1.48	1.16
D3	Position [cm^{-1}]	1447	1446
	FWHM [cm^{-1}]	46	67
	A [cm^2]	323	12404
	A_{D3+D4}/A_{total}	0.07	0.17
D4	Position [cm^{-1}]	1195	1180
	FWHM [cm^{-1}]	161	219
	A [cm^2]	3648	56113
	A_{D4+D3}/A_{total}	0.07	0.17

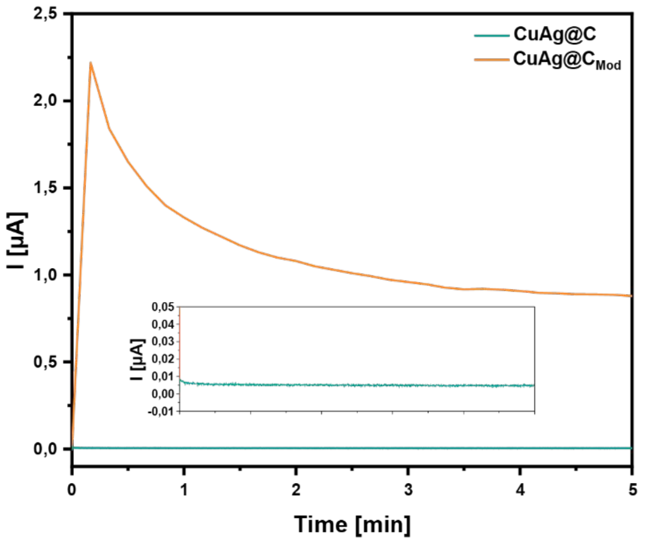

Figure S4 Current vs. time curves of CuAg@C and CuAg@C_{Mod} measured in R2032 coin cells. Symmetric cell setup containing stainless steel||sample||stainless steel was constructed without any electrolyte. The current vs. time curves were recorded at 2 V for five minutes at 25 ºC.

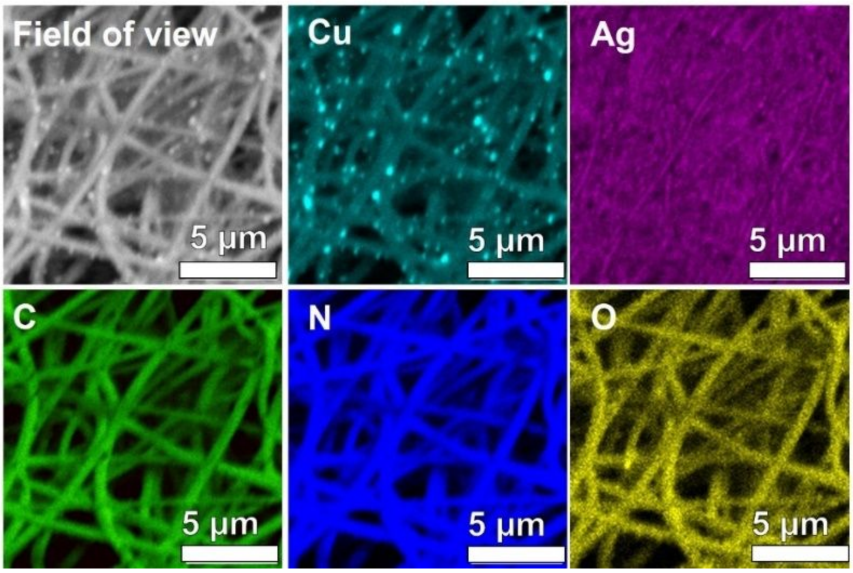

Figure S5 SEM-EDS images of CuAg@C showing the field of view and the elemental distribution of Cu, Ag, C, N and O. The different particle resolutions of Cu and Ag are a result of the corresponding excitation volume (Cu L, 0.93 keV and Ag L, 2.96 keV).

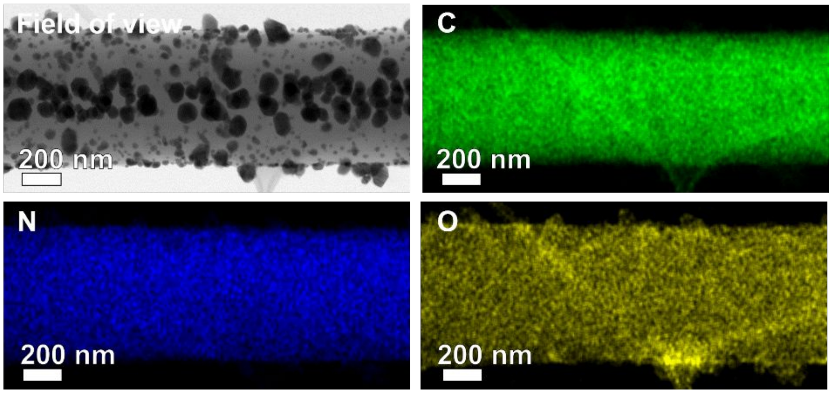

Figure S6 STEM-EDX maps of CuAg@C showing the field of view and the elemental distribution of C, N and O.

Table S2 Measurement details for the different XPS scans.

ID	Scans	Dwell time [ms]	Pass energy [eV]	Range [eV]
Survey	10	10	200	10-1350
C 1s	5	50	50	279-305
O 1s	10	50	50	525-545
Li 1s	20	50	50	51-70
N 1s	10	50	50	392-410
Cu 2p	10	50	50	925-965
Ag 3d	10	50	50	360-380

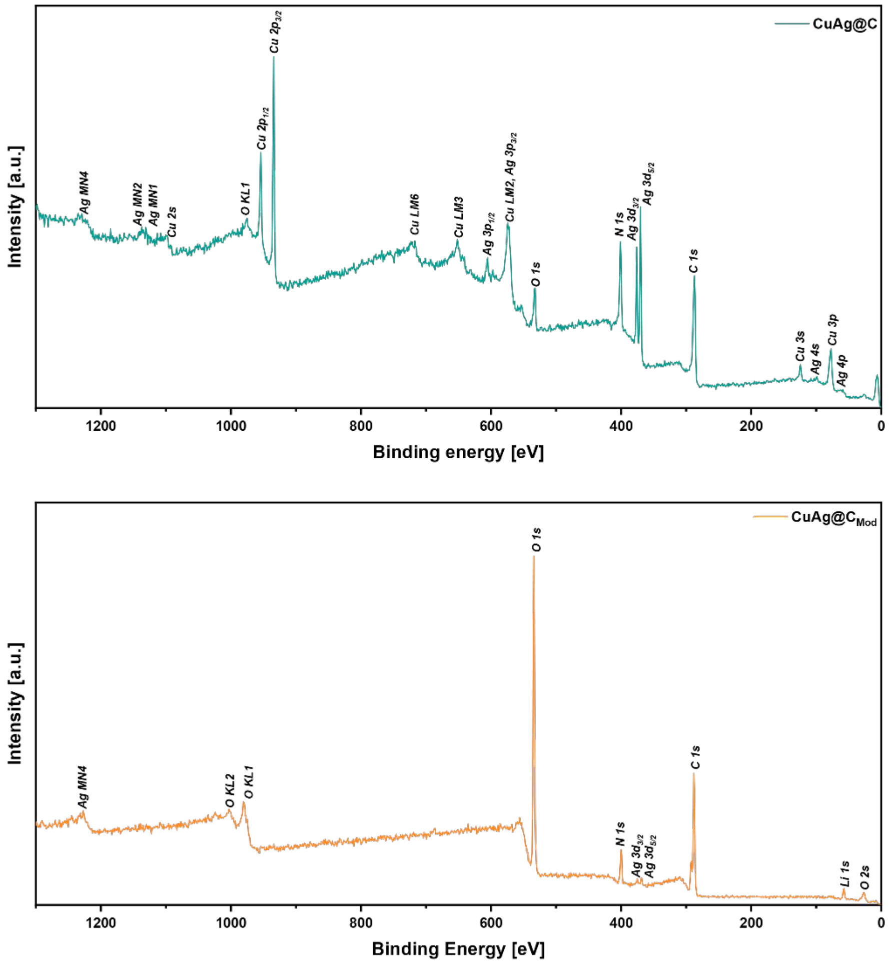

Figure S7 Survey measurement of the XPS spectrum for CuAg@C (top) and CuAg@C_{Mod} (bottom). Measurements details are listed in **Table S2**.

Table S3 Detailed fitting parameters (param.) of C 1s for CuAg@C from the first analyzed point with corrected and uncorrected binding energies (BE). The Ag $3d_{5/2}$ signal is set to 368.2 eV.

**Peak fit table: C 1s scan**	**C-C/C-H**	**CN /C-O**	**C=O**
Uncorrected BE [eV]	284.7	286.4	289.5
Corrected BE [eV]	284.7	286.4	289.5
Height CPS	21959.1	8835.5	1195.2
Height ratio	1.0	0.4	0.05
Area CPS [eV]	37573.1	17558.1	2490.9
Area ratio	1.0	0.5	0.07
FWHM fit param. [eV]	1.4	1.9	2.0
L/G mix [%] convolve	30.0	30.0	30.0
Tail mix [%]	84.9	100.0	100.0
Tail height [%]	0	0	0
Tail exponent	0.04	0	0
Atomic%	40.6	19.0	2.7

Table S4 Detailed fitting param. of O 1s for CuAg@C from the first analyzed point with corrected and uncorrected BEs. The Ag $3d_{5/2}$ signal is set to 368.2 eV.

**Peak fit table: O 1s scan**	**C=O**	**C-O**
Uncorrected BE [eV]	530.6	532.8
Corrected BE [eV]	530.6	532.8
Height CPS	3232.2	1475.5
Height ratio	1.0	0.5
Area CPS [eV]	6735.8	3074.8
Area ratio	1.0	0.5
FWHM fit param. [eV]	2	2
L/G mix [%] convolve	30.0	30
Tail mix [%]	100.0	100
Tail height [%]	0	0
Tail exponent	0	0
Atomic%	3.0	1.4

Table S5 Detailed fitting param. of N 1s for CuAg@C from the first analyzed point with corrected and uncorrected BEs. The Ag $3d_{5/2}$ signal is set to 368.2 eV.

**Peak fit table: N 1s scan**	**Pyridinic N**	**Pyrrolic N**
Uncorrected BE [eV]	398.6	400.5
Corrected BE [eV]	398.6	400.5
Height CPS	11928.1	2837.3
Height ratio	1.0	0.2
Area CPS [eV]	20524.8	5473.3
Area ratio	1.0	0.3
FWHM fit param. [eV]	1.7	1.9
L/G mix [%] convolve	30.0	30.0
Tail mix [%]	100.0	100.0
Tail height [%]	0	0
Tail exponent	0	0
Atomic%	14.3	3.8

Table S6 Detailed fitting param. of Ag 3d for CuAg@C from the first analyzed point with corrected and uncorrected BEs. The Ag $3d_{5/2}$ signal is set to 368.2 eV.

**Peak fit table: Ag 3d scan**	**Ag (doublet)**	
Uncorrected BE [eV]	368.2	374.2
Corrected BE [eV]	368.2	374.2
Height CPS	94560.3	65464.8
Height ratio	1.0	0.7
Area CPS [eV]	89172.3	61734.7
Area ratio	1.0	0.7
FWHM fit param. [eV]	0.9	0.9
L/G mix [%] convolve	30.0	30.0
Tail mix [%]	100.0	100.0
Tail height [%]	0	0
Tail exponent	0	0
Atomic%	7.8	-

Table S7 Detailed fitting param. of Cu 2p for CuAg@C from the first analyzed point with corrected and uncorrected BEs. The Ag $3d_{5/2}$ signal is set to 368.2 eV.

Peak fit table: Cu 2p scan	Cu (doublet)	
Uncorrected BE [eV]	932.5	952.3
Corrected BE [eV]	932.5	952.3
Height CPS	42174.8	21831.1
Height ratio	1.0	0.5
Area CPS [eV]	69908.6	36187.0
Area ratio	1.0	0.5
FWHM fit param. [eV]	1.6	1.6
L/G mix [%] convolve	30.0	30.0
Tail mix [%]	100.0	100.0
Tail height [%]	0	0
Tail exponent	0	0
Atomic%	7.4	-

Table S8 Detailed fitting param. of C 1s for CuAg@C_{Mod} from the first analyzed point with corrected and uncorrected BEs. The C=C/C-C signal is set to 285.0 eV.

**Peak fit table: C1s scan**	**C-Li**	**C-C/C-H**	**C-O/CN**	**RCO_2Li**	**$ROCO_2Li$**
Uncorrected BE [eV]	285.0	286.9	288.4	290.6	292.1
Corrected BE [eV]	283.3	285.0	286.5	288.7	290.2
Height CPS	1003.5	27113.4	3694.3	1206.9	1227.7
Height ratio	0.04	1.0	0.1	0.04	0.05
Area CPS [eV]	1018.9	41327.0	6214.7	2515.0	1551.8
Area ratio	0.04	1.0	0.2	0.06	0.04
FWHM fit param. [eV]	0.9	1.5	1.6	2.0	1.2
L/G mix [%] convolve	30.0	30.0	30.0	30.0	30.0
Tail mix [%]	100.0	100.0	100.0	100.0	100.0
Tail height [%]	0	0	0	0	0
Tail exponent	0	0	0	0	0
Atomic%	0.8	31.0	4.7	1.9	1.2

Table S9 Detailed fitting param. of O 1s for CuAg@C_{Mod} from the first analyzed point with corrected and uncorrected BEs. The C=C/C-C signal is set to 285.0 eV.

**Peak fit table: O1s scan**	**Li_2O**	**$RCO_2Li/ROCO_2Li$**
Uncorrected BE [eV]	530.6	533.2
Corrected BE [eV]	528.7	531.3
Height CPS	2329.8	33291.4
Height ratio	0.1	1.0
Area CPS [eV]	4855.2	69378.4
Area ratio	0.1	1.0
FWHM fit param. [eV]	1.0	2.4
L/G mix [%] convolve	30.0	30.0
Tail mix [%]	100.0	100.0
Tail height [%]	0	0
Tail exponent	0	0
Atomic%	1.5	21.5

Table S10 Detailed fitting param. of Li 1s for CuAg@C_{Mod} from the first analyzed point with corrected and uncorrected BEs. The C=C/C-C signal is set to 285.0 eV.

**Peak fit table: Li 1s scan**	**Li_xR**
Uncorrected BE [eV]	57.2
Corrected BE [eV]	55.3
Height CPS	1639.9
Height ratio	1.0
Area CPS [eV]	3347.8
Area ratio	1.0
FWHM fit param. [eV]	2.0
L/G mix [%] convolve	30.0
Tail mix [%]	100.0
Tail height [%]	0
Tail exponent	0
Atomic%	36.0

Table S11 Detailed fitting param. of N 1s for $CuAg@C_{Mod}$ from the first analyzed point with corrected and uncorrected BEs. The C=C/C-C signal is set to 285.0 eV.

**Peak fit table: N 1s scan**	**Pyridinic N**	**Pyrrolic N**
Uncorrected BE [eV]	399.2	400.8
Corrected BE [eV]	397.3	398.9
Height CPS	845.4	751.5
Height ratio	1.0	0.9
Area CPS [eV]	1621.0	1132.6
Area ratio	1.0	0.7
FWHM fit param. [eV]	1.8	1.5
L/G mix [%] convolve	30.0	30.0
Tail mix [%]	100.0	100.0
Tail height [%]	0	0
Tail exponent	0	0
Atomic%	0.8	0.6

Table S12 Detailed fitting param. of Ag 3d for CuAg@C_{Mod} from the first analyzed point with corrected and uncorrected BEs. The C=C/C-C signal is set to 285.0 eV.

**Peak fit table: Ag 3d scan**	**Ag (doublet)**	
Uncorrected BE [eV]	368.4	374.4
Corrected BE [eV]	366.5	372.5
Height CPS	2115.4	1464.5
Height ratio	1.0	0.7
Area CPS [eV]	1818.1	1258.7
Area ratio	1.0	0.7
FWHM fit param. [eV]	0.8	0.8
L/G mix [%] convolve	30.0	30.0
Tail mix [%]	100.0	100.0
Tail height [%]	0	0
Tail exponent	0	0
Atomic%	0.1	-

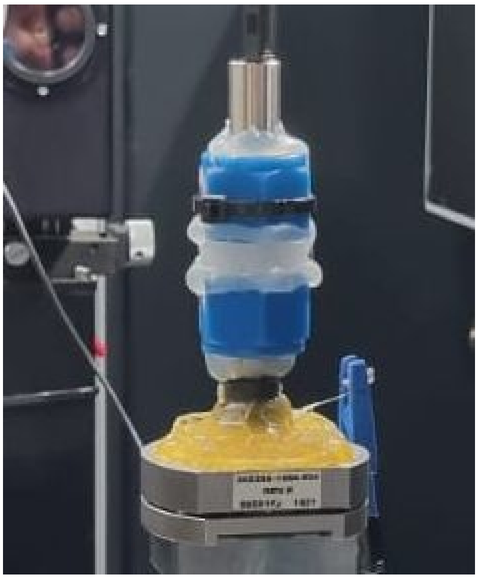

Figure S8 Standard perfluoroalkoxy alkane Swagelok-type cell, with a cell configuration of Cu foil/CuAg@C_{Mod}||electrolyte||Li, for in-situ X-ray CT measurements. Liquid glue was used to seal the cell airtight. A zinc wire was used at the bottom to connect the cell to the potentiostat. A direct connection was impossible since the cell had to be attached to the sample holder.

Table S13 Calculated average thickness of $CuAg@C_{Mod}$ after each plating and stripping step with the corresponding standard derivation. The listed values correspond to the *in-situ* X-ray CT measurement.

Cycling State	Fiber thickness [µm]	Standard derivation [µm]
**1.** Plating	164	8
**1.** Stripping	143	9
**2.** Plating	165	8
**2.** Stripping	145	8
**3.** Plating	164	8
**3.** Stripping	144	9
**4.** Plating	164	8
**4.** Stripping	144	9

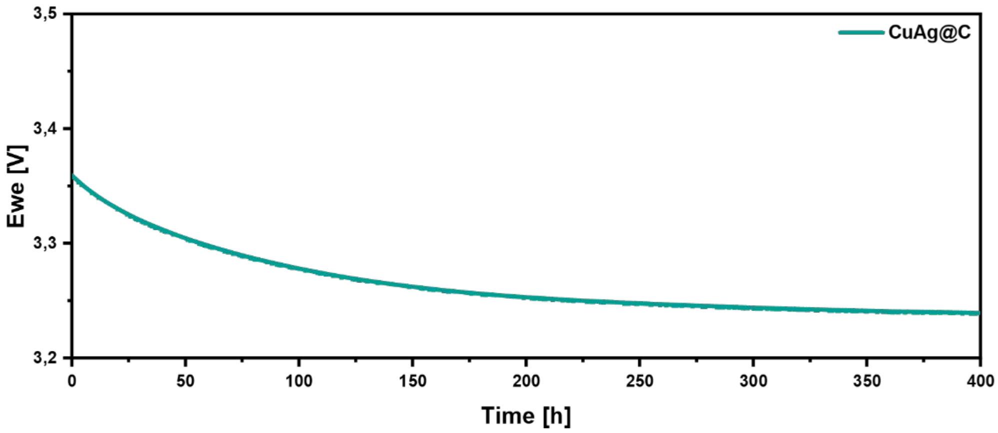

Figure S9 Charge curve of CuAg@C tested in Cu foil/CuAg@C‖electrolyte‖LFP cells for the initial cycle.

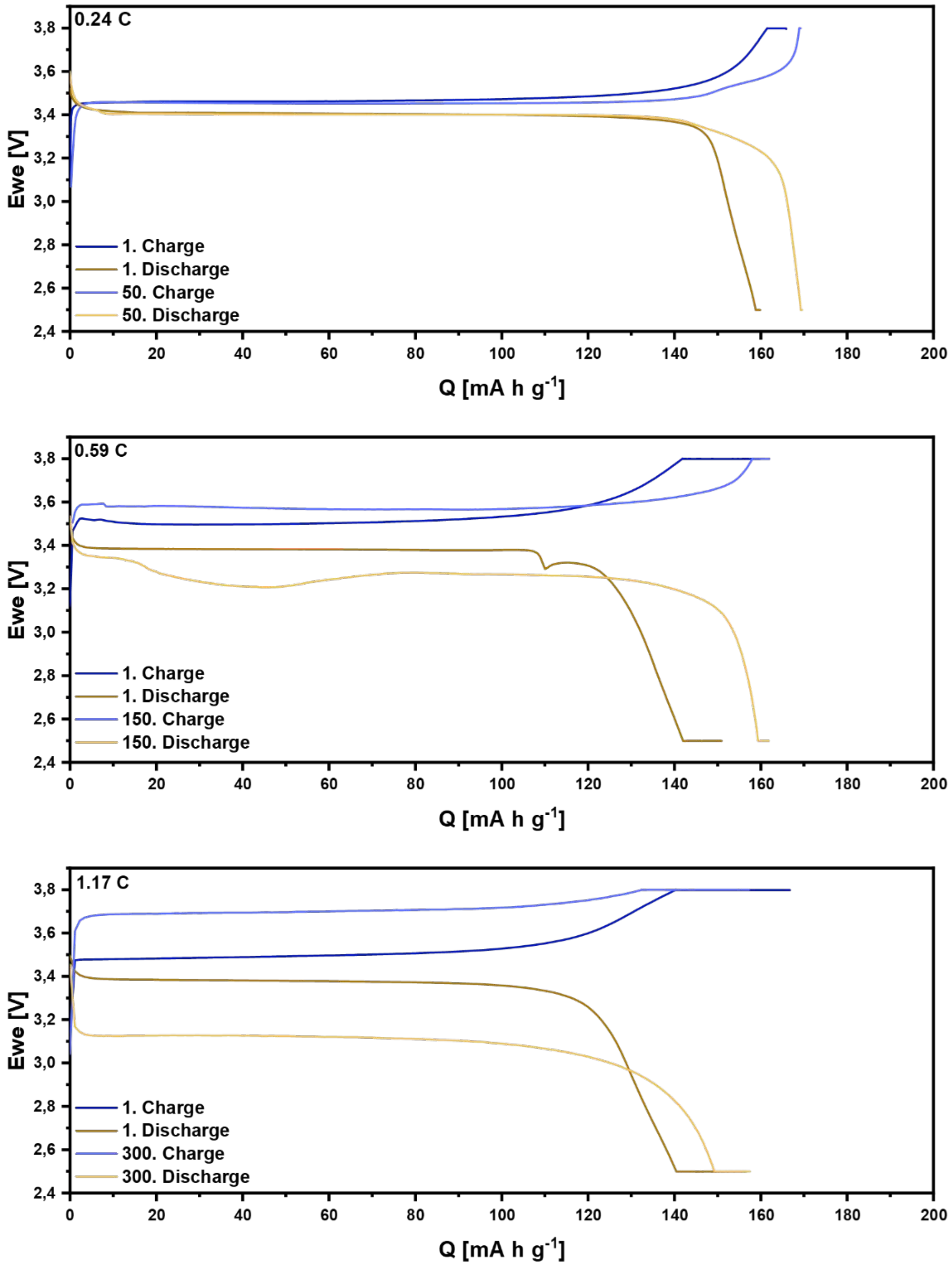

Figure S10 Charging-discharging curves of CuAg@C_{Mod} tested in Cu foil/CuAg@C_{Mod}||electrolyte||LFP cells for the initial and the last cycles at 0.24 C, 0.59 C and 1.17 C, respectively.

Table S14 Overview of different *zero-excess* Li metal batteries known in the literature.

Cell design	CE [%]	Cycles	Capacity retention [%]	Reference
Cu||$LiFePO_4$	99	50	60	1
Cu||NMC	23			2
CP||NMC_{811}	98.6	50	90	3
Cu/Al_2O_3||LTO	98.90	120	80	4
Cu/Al_2O_3||NMC	96.7	50	70	4
Cu/Li-SiO_x||LFP	99.9	100	93.5	5
Cu/Graphene oxide||NMC	98	50	44	6
AOP@Cu||NMC	96.1	82	30	7
CFs@Au-Li||S	99.2	100	56	8
Li–CMN||$LiFePO_4$	99.6	400	64	9
Cu-Ag @ Li||NMC	98.48	100	85	10
MLG layer||$LiFePO_4$	99.51	100	61.3	11
Cu3N||$LiFePO_4$	97.6	50	30	12
Cu–Sn||NCA	93	80	→0	13
Cu||LFP	98.9	50	50	14
Cu||Li_2S	95-99	150	40	15
Au/Cu||Li_2S	95-99	150	53	15
Cu||NMC_{532}		50	50	16
Cu||NMC_{111}		90	80	17
Ag@carbon paper	98	110	110	18
Al_2O_3@PAN||NMC	97	82	82	19
Cu-CNTS||$LiFePO_4$	95-99	100	69.4	20
NGCS||Li	99.35	220		21
Cu||$LiFePO_4$	99.70	100	44.5	22
Cu@PEO||$LiFePO_4$	98.6	200	30	23
$LiNO_3$/CB + PVDF||$LiFePO_4$	99.3	100		24
Cu@Si-PAN||$LiNi_{0.5}Mn_{1.5}O_4$	99	120		25
Cu +Ag PCP||$LiFePO_4$	99.8	200	72	26
Au-3D Cu||$LiFePO_4$	close to 100	100		27
Cu||$LiFePO_4$	99.2	100	44.5	28
**This work**	**~100**	**300**	**92.6**	

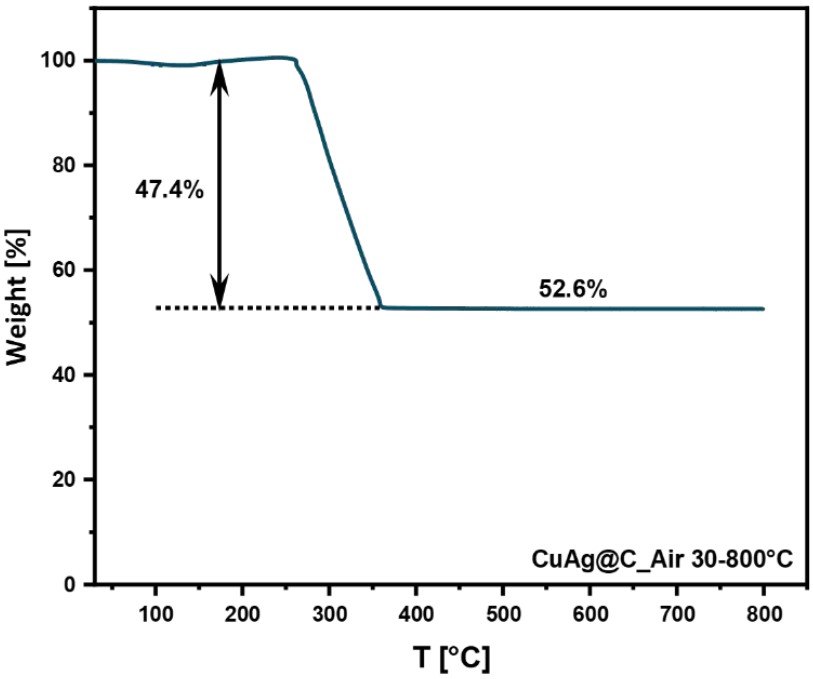

Figure S11 Thermogravimetric analysis (TGA) curve of CuAg@C measured under atmosphere from 30 to 800 °C in air.

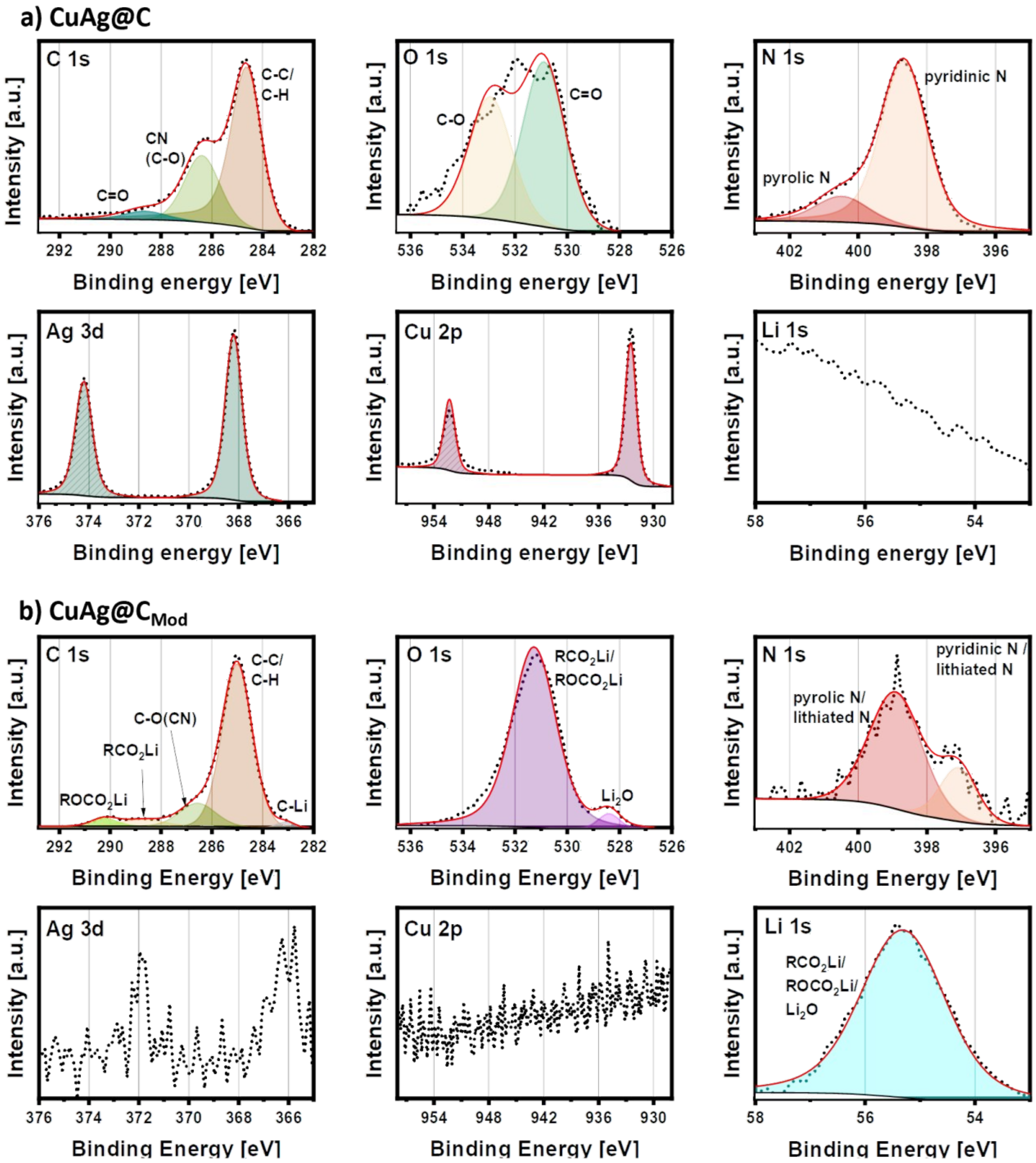

Figure S12 C 1s, O 1s, N 1s, Ag 3d, Cu 2p and Li 1s XPS spectra of a) CuAg@C and b) CuAg@C$_{Mod}$ for the second measured point. The measurement parameters can be found in **Table S2**. All spectra are normalized, with the highest signal in each spectrum set to 1. Due to the normalization, the measurement of Li 1s for CuAg@C and the Cu 2p for CuAg@C$_{Mod}$ shows a high background noise. For comparison, the Ag 3d$_{5/2}$ signal is set to 368.2 eV and used as the reference for CuAg@C, while for CuAg@C$_{Mod}$ the C-C/C-H signal in the C 1s is set to 285.0 eV.

Table S15 Detailed fitting parameters (param.) of C 1s for CuAg@C from the second analyzed point with corrected and uncorrected binding energies (BE). The Ag $3d_{5/2}$ signal is set to 368.2 eV.

**Peak fit table: C 1s scan**	**C-C/C-H**	**CN /C-O**	**C=O**
Uncorrected BE [eV]	284.6	286.3	289.3
Corrected BE [eV]	284.6	286.3	289.3
Height CPS	23434.5	9780.1	672.5
Height ratio	1.0	0.4	0.03
Area CPS [eV]	39100.3	22207.7	1577.3
Area ratio	1.0	0.6	0.04
FWHM fit param. [eV]	1.3	1.9	2.0
L/G mix [%] convolve	20.0	30.0	30.0
Tail mix [%]	84.9	100.0	100.0
Tail height [%]	0	0	0
Tail exponent	0.04	0	0
Atomic%	37.6	21.4	1.5

Table S16 Detailed fitting param. of O 1s for CuAg@C from the second analyzed point with corrected and uncorrected BEs. The Ag $3d_{5/2}$ signal is set to 368.2 eV.

Peak fit table: O 1s scan	C=O	C-O
Uncorrected BE [eV]	530.9	532.8
Corrected BE [eV]	530.6	532.8
Height CPS	4847.6	3593.4
Height ratio	1.0	0.7
Area CPS [eV]	10102.3	7488.6
Area ratio	1.0	0.7
FWHM fit param. [eV]	2	2
L/G mix [%] convolve	30.0	30
Tail mix [%]	100.0	100
Tail height [%]	0	0
Tail exponent	0	0
Atomic%	4.0	3.0

Table S17 Detailed fitting param. of N 1s for CuAg@C from the second analyzed point with corrected and uncorrected BEs. The Ag $3d_{5/2}$ signal is set to 368.2 eV.

Peak fit table: N 1s scan	Pyridinic N	Pyrrolic N
Uncorrected BE [eV]	398.7	400.5
Corrected BE [eV]	398.6	400.5
Height CPS	12840.1	2001.6
Height ratio	1.0	0.2
Area CPS [eV]	24157.9	4234.1
Area ratio	1.0	0.2
FWHM fit param. [eV]	1.6	1.8
L/G mix [%] convolve	30.0	30.0
Tail mix [%]	100.0	100.0
Tail height [%]	0	0
Tail exponent	0	0
Atomic%	14.9	2.6

Table S18 Detailed fitting param. of Ag 3d for CuAg@C from the second analyzed point with corrected and uncorrected BEs. The Ag $3d_{5/2}$ signal is set to 368.2 eV.

**Peak fit table: Ag 3d scan**	**Ag (doublet)**	
Uncorrected BE [eV]	368.2	374.2
Corrected BE [eV]	368.2	374.2
Height CPS	80498.8	55729.9
Height ratio	1.0	0.7
Area CPS [eV]	76859.9	53210.7
Area ratio	1.0	0.7
FWHM fit param. [eV]	0.8	0.8
L/G mix [%] convolve	30.0	30.0
Tail mix [%]	100.0	100.0
Tail height [%]	0	0
Tail exponent	0	0
Atomic%	5.9	-

Table S19 Detailed fitting param. of Cu 2p for CuAg@C from the second analyzed point with corrected and uncorrected BEs. The Ag $3d_{5/2}$ signal is set to 368.2 eV.

**Peak fit table: Cu 2p scan**	**Cu (doublet)**	
Uncorrected BE [eV]	932.5	952.4
Corrected BE [eV]	932.5	952.3
Height CPS	54029.2	27967.3
Height ratio	1.0	0.5
Area CPS [eV]	93295.7	48292.9
Area ratio	1.0	0.5
FWHM fit param. [eV]	1.5	1.5
L/G mix [%] convolve	30.0	30.0
Tail mix [%]	100.0	100.0
Tail height [%]	0	0
Tail exponent	0	0
Atomic%	8.7	-

Table S20 Detailed fitting param. of C 1s for CuAg@C_{Mod} from the second analyzed point with corrected and uncorrected BEs. The C=C/C-C signal is set to 285.0 eV.

**Peak fit table: C 1s scan**	**C-Li**	**C-C/C-H**	**C-O/CN**	**RCO_2Li**	**$ROCO_2Li$**
Uncorrected BE [eV]	284.9	286.8	288.2	290.6	292.1
Corrected BE [eV]	283.1	285.0	286.4	288.7	290.2
Height CPS	893.1	23683.5	3812.6	1004.2	1187.2
Height ratio	0.04	1.0	0.2	0.04	0.05
Area CPS [eV]	827.3	35009.0	7945.3	2092.6	1556.3
Area ratio	0.04	1.0	0.23	0.06	0.04
FWHM fit param. [eV]	0.9	1.4	2.0	2.0	1.3
L/G mix [%] convolve	30.0	30.0	30.0	30.0	30.0
Tail mix [%]	100.0	100.0	100.0	100.0	100.0
Tail height [%]	0	0	0	0	0
Tail exponent	0	0	0	0	0
Atomic%	0.7	28.2	6.4	1.7	1.3

Table S21 Detailed fitting param. of O 1s for CuAg@C_{Mod} from the second analyzed point with corrected and uncorrected BEs. The C=C/C-C signal is set to 285.0 eV.

**Peak fit table: O 1s scan**	**Li_2O**	**$RCO_2Li/ROCO_2Li$**
Uncorrected BE [eV]	530.2	533.1
Corrected BE [eV]	528.4	531.3
Height CPS	2604.8	29236.8
Height ratio	0.09	1.0
Area CPS [eV]	3053.6	68575.5
Area ratio	0.04	1.0
FWHM fit param. [eV]	1.0	2.0
L/G mix [%] convolve	30.0	30.0
Tail mix [%]	100.0	100.0
Tail height [%]	0	0
Tail exponent	0	0
Atomic%	1.0	22.8

Table S22 Detailed fitting param. of Li 1s for CuAg@C_{Mod} from the second analyzed point with corrected and uncorrected BEs. The C=C/C-C signal is set to 285.0 eV.

**Peak fit table: Li 1s scan**	**Li_xR**
Uncorrected BE [eV]	57.1
Corrected BE [eV]	55.3
Height CPS	1488.9
Height ratio	1.0
Area CPS [eV]	3182.6
Area ratio	1.0
FWHM fit param. [eV]	1.8
L/G mix [%] convolve	30.0
Tail mix [%]	100.0
Tail height [%]	0
Tail exponent	0
Atomic%	36.7

Table S23 Detailed fitting param. of N 1s for CuAg@C_{Mod} from the second analyzed point with corrected and uncorrected BEs. The C=C/C-C signal is set to 285.0 eV.

**Peak fit table: N 1s scan**	**Pyridinic N**	**Pyrrolic N**
Uncorrected BE [eV]	398.9	400.7
Corrected BE [eV]	397.1	398.9
Height CPS	355.2	770.1
Height ratio	0.5	1.0
Area CPS [eV]	490.8	1511.7
Area ratio	0.3	1.0
FWHM fit param. [eV]	1.3	1.9
L/G mix [%] convolve	30.0	30.0
Tail mix [%]	100.0	100.0
Tail height [%]	0	0
Tail exponent	0	0
Atomic%	0.3	0.8

Table S24 Detailed fitting param. of Ag 3d for CuAg@C_{Mod} from the second analyzed point with corrected and uncorrected BEs. The C=C/C-C signal is set to 285.0 eV.

Peak fit table: Ag 3d scan	Ag (doublet)	
Uncorrected BE [eV]	368.5	374.5
Corrected BE [eV]	366.7	372.7
Height CPS	4520.2	3129.4
Height ratio	1.0	0.7
Area CPS [eV]	4246.6	2940.0
Area ratio	1.0	0.7
FWHM fit param. [eV]	0.8	0.8
L/G mix [%] convolve	30.0	30.0
Tail mix [%]	100.0	100.0
Tail height [%]	0	0
Tail exponent	0	0
Atomic%	0.3	-

References

(1) Qian, J.; Adams, B. D.; Zheng, J.; Xu, W.; Henderson, W. A.; Wang, J.; Bowden, M. E.; Xu, S.; Hu, J.; Zhang, J.-G. Anode-Free Rechargeable Lithium Metal Batteries. *Adv. Funct. Mat.* **2016**, *26*, 7094–7102.

(2) Woo, J.-Jung.; Maroni, Victor. A.; Liu, G.; Vaughey, John T.; Gosztola, David J.; Amine, K.; Zhang, Z. Symmetrical Impedance Study on Inactivation Induced Degradation of Lithium Electrodes for Batteries Beyond Lithium-Ion. *J. Electrochem. Soc.* **2014**, *161*, A827-A830.

(3) Kwon, H.; Lee, J.-H.; Roh, Y.; Baek, J.; Shin, D. J.; Yoon, J. K.; Ha, H. J.; Kim, J. Y.; Kim, H.-T. An Electron-Deficient Carbon Current Collector for Anode-Free Li-metal Batteries. *Nat. Commun.* **2021**, *12*, 5537.

(4) Tu, Z.; Zachman, M. J.; Choudhury, S.; Khan, K. A.; Zhao, Q.; Kourkoutis, L. F.; Archer, L. A. Stabilizing Protic and Aprotic Liquid Electrolytes at High-Bandgap Oxide Interphases. *Chem. Mat.* **2018**, *30*, 5655–5662.

(5) Chen, W.; Salvatierra, R. V.; Ren, M.; Chen, J.; Stanford, M. G.; Tour, J. M. Laser-Induced Silicon Oxide for Anode-Free Lithium Metal Batteries. *Adv. Mat.* **2020**, *32*, e2002850.

(6) Wondimkun, Z. T.; Beyene, T. T.; Weret, M. A.; Sahalie, N. A.; Huang, C.-J.; Thirumalraj, B.; Jote, B. A.; Wang, D.; Su, W.-N.; Wang, C.-H.; Brunklaus, G.; Winter, M.; Hwang, B.-J. Binder-Free Ultra-thin Graphene Oxide as an Artificial Solid Electrolyte Interphase for Anode-Free Rechargeable Lithium Metal Batteries. *J. Power Sources* **2020**, *450*, 227589.

(7) Sahalie, N. A.; Wondimkun, Z. T.; Su, W.-N.; Weret, M. A.; Fenta, F. W.; Berhe, G. B.; Huang, C.-J.; Hsu, Y.-C.; Hwang, B. J. Multifunctional Properties of Al 2 O 3 /Polyacrylonitrile Composite Coating on Cu to Suppress Dendritic Growth in Anode-Free Li-Metal Battery. *ACS Appl. Energy Mat.* **2020**, *3*, 7666–7679.

(8)Xiang, J.; Yuan, L.; Shen, Y.; Cheng, Z.; Yuan, K.; Guo, Z.; Zhang, Y.; Chen, X.; Huang, Y. Improved Rechargeability of Lithium Metal Anode *via* Controlling Lithium-Ion Flux. *Adv. Energy Mat.* **2018**, *8,* 1802352.

(9) Ye, H.; Xin, S.; Yin, Y.-X.; Guo, Y.-G. Advanced Porous Carbon Materials for High-Efficient Lithium Metal Anodes. *Adv. Energy Mat.* **2017**, *7*, 1700530.

(10) Cui, S.; Zhai, P.; Yang, W.; Wei, Y.; Xiao, J.; Deng, L.; Gong, Y. Large-Scale Modification of Commercial Copper Foil with Lithiophilic Metal Layer for Li Metal Battery. *Small (Weinheim an der Bergstrasse, Germany)* **2020**, *16*, e1905620.

(11) Assegie, A. A.; Cheng, J.-H.; Kuo, L.-M.; Su, W.-N.; Hwang, B.-J. Polyethylene Oxide Film Coating Enhances Lithium Cycling Efficiency of an Anode-Free Lithium-Metal Battery. *Nanoscale* **2018**, *10*, 6125–6138.

(12) Li, Q.; Pan, H.; Li, W.; Wang, Y.; Wang, J., Zheng, J.; Yu, X.; Li, H.; Chen, L. Homogeneous Interface Conductivity for Lithium Dendrite-Free anode. *ACS Energy Lett.* **2018**, *3*, 2259-2266.

(13) Zhang, S. S.; Fan, X.; Wang, C. A Tin-Plated Copper Substrate for Efficient Cycling of Lithium Metal in an Anode-Free Rechargeable Lithium Battery. *Electrochimica Acta* **2017**, *258*, 1201–1207.

(14) Beyene, Tamene T.; Bezabh, H. Kassa; Weret, Misganaw A.; Hagos, Teklay M.; Huang, Chen J.; Wang, Chia-H.; Su, Wei-N.; Dai, H.; Hwang, Bing-J. Concentrated Dual-Salt Electrolyte to Stabilize Li Metal and Increase Cycle Life of Anode Free Li-Metal Batteries. *J. Electrochem. Soc.* **2019**, *166*, A1501-A1509.

(15) Chen, J.; Xiang, J.; Chen, X.; Yuan, L.; Li, Z.; Huang, Y. Li_2S-Based Anode-Free Full Batteries with Modified Cu Current Collector. *Energy Storage Mat.* **2020**, *30*, 179–186.

(16) Louli, A. J.; Genovese, M.; Weber, R.; Hames, S. G.; Logan, E. R.; Dahn, J. R. Exploring the Impact of Mechanical Pressure on the Performance of Anode-Free Lithium Metal Cells. *J. Electrochem. Soc.* **2019**, *166*, A1291-A1299.

(17) Weber, R.; Genovese, M.; Louli, A. J.; Hames, S.; Martin, C.; Hill, I. G.; Dahn, J. R. Long Cycle Life and Dendrite-Free Lithium Morphology in Anode-Free Lithium Pouch Cells Enabled by a Dual-Salt Liquid Electrolyte. *Nat. Energy* **2019**, *4*, 683–689.

(18) Liu, H.; Yue, X.; Xing, X.; Yan, Q.; Huang, J.; Petrova, V.; Zhou, H.; Liu, P. A Scalable 3D Lithium Metal Anode. *Energy Storage Mat.* **2019**, *16*, 505–511.

(19) Sahalie, N. A.; Wondimkun, Z. T.; Su, W.-N.; Weret, M. A.; Fenta, F. W.; Berhe, G. B.; Huang, C.-J.; Hsu, Y.-C.; Hwang, B. J. Multifunctional Properties of Al_2O_3 /Polyacrylonitrile Composite Coating on Cu to Suppress Dendritic Growth in Anode-Free Li-Metal Battery. *ACS Appl. Energy Mat.* **2020**, *3*, 7666–7679.

(20) Shan, C.; Qin, Z.; Xie, Y.; Meng, X.; Chen, J.; Chang, Y.; Zang, R.; Wan, L.; Huang, Y. Cu-CNTs Current Collector Fabricated by Deformation-Driven Metallurgy for Anode-Free Li Metal Batteries. *Carbon* **2023**, *204*, 367–376.

(21) Hou, G.; Ren, X.; Ma, X.; Le Zhang; Zhai, W.; Ai, Q.; Xu, X.; Zhang, L.; Si, P.; Feng, J.; Ding, F.; Ci, L. Dendrite-Free Li Metal Anode Enabled by a 3D Free-Standing Lithiophilic Nitrogen-Enriched Carbon Sponge. *J. Power Sources* **2018**, *386*, 77–84.

(22) Lin, Y.; Chen, J.; Zhang, H.; Wang, J. *In-situ* Construction of High-Mechanical-Strength and Fast-Ion-Conductivity Interphase for Anode-Free Li Battery. *J. Energy Chemistry* **2023**, *80*, 207–214.

(23) Assegie, A. A.; Cheng, J.-H.; Kuo, L.-M.; Su, W.-N.; Hwang, B.-J. Polyethylene Oxide Film Coating Enhances Lithium Cycling Efficiency of an Anode-Free Lithium-Metal Battery. *Nanoscale* **2018**, *10*, 6125–6138.

(24) Li, X.; Yang, G.; Zhang, S.; Wang, Z.; Chen, L. Improved Lithium Deposition on Silver Plated Carbon Fiber Paper. *Nano Energy* **2019**, *66*, 104144.

(25) Liang, P.; Sun, H.; Huang, C.-L.; Zhu, G.; Tai, H.-C.; Li, J.; Wang, F.; Wang, Y.; Huang, C.-J.; Jiang, S.-K.; Lin, M.-C.; Li, Y.-Y.; Hwang, B.-J.; Wang, C.-A.; Dai, H. A Nonflammable High-Voltage 4.7 V Anode-Free Lithium Battery. *Adv. Mat.* **2022**, *34*, e2207361.

(26) Pyo, S.; Ryu, S.; Gong, Y. J.; Cho, J.; Yun, H.; Kim, H.; Lee, J.; Min, B.; Choi, Y.; Yoo, J.; Kim, Y. S. Lithiophilic Wetting Agent Inducing Interfacial Fluorination for Long-Lifespan Anode-Free Lithium Metal Batteries. *Adv. Energy Mat.* **2023**, *13,* 2203573.

(27) Kim, E.; Choi, W.; Ryu, S.; Yun, Y.; Jo, S.; Yoo, J. Effect of 3D Lithiophilic Current Collector for Anode-Free Li ion Batteries. *J. Alloys Compd*. **2023**, *966*, 171393.

(28) Lin, Y.; Chen, J.; Zhang, H.; Wang, J. *In-situ* Construction of High-Mechanical-Strength and Fast-Ion-Conductivity Interphase for Anode-Free Li Battery. *J. Energy Chem.* **2023**, *80*, 207–214.
